# Nanoparticles incorporated hydrogels for delivery of antimicrobial agents: developments and trends

**DOI:** 10.1039/d4ra00631c

**Published:** 2024-04-25

**Authors:** Naveed Ahmad, Syed Nasir Abbas Bukhari, Muhammad Ajaz Hussain, Hasan Ejaz, Muhammad Usman Munir, Muhammad Wahab Amjad

**Affiliations:** a Department of Pharmaceutics, College of Pharmacy, Jouf University Sakaka 72388 Aljouf Saudi Arabia nakahmad@ju.edu.sa naveedpharmacist@yahoo.com; b Department of Pharmaceutical Chemistry, College of Pharmacy, Jouf University Sakaka 72388 Aljouf Saudi Arabia; c Centre for Organic Chemistry, School of Chemistry, University of the Punjab Lahore 54590 Pakistan; d Department of Clinical Laboratory Sciences, College of Applied Medical Sciences, Jouf University Sakaka 72388 Aljouf Saudi Arabia; e Australian Institute for Bioengineering & Nanotechnology, The University of Queensland Brisbane Queens-land 4072 Australia; f 6 Center for Ultrasound Molecular Imaging and Therapeutics, School of Medicine, University of Pittsburgh 15213 Pittsburgh Pennsylvania USA

## Abstract

The prevention and treatment of microbial infections is an imminent global public health concern due to the poor antimicrobial performance of the existing antimicrobial regime and rapidly emerging antibiotic resistance in pathogenic microbes. In order to overcome these problems and effectively control bacterial infections, various new treatment modalities have been identified. To attempt this, various micro- and macro-molecular antimicrobial agents that function by microbial membrane disruption have been developed with improved antimicrobial activity and lesser resistance. Antimicrobial nanoparticle-hydrogels systems comprising antimicrobial agents (antibiotics, biological extracts, and antimicrobial peptides) loaded nanoparticles or antimicrobial nanoparticles (metal or metal oxide) constitute an important class of biomaterials for the prevention and treatment of infections. Hydrogels that incorporate nanoparticles can offer an effective strategy for delivering antimicrobial agents (or nanoparticles) in a controlled, sustained, and targeted manner. In this review, we have described an overview of recent advancements in nanoparticle–hydrogel hybrid systems for antimicrobial agent delivery. Firstly, we have provided an overview of the nanoparticle hydrogel system and discussed various advantages of these systems in biomedical and pharmaceutical applications. Thereafter, different hybrid hydrogel systems encapsulating antibacterial metal/metal oxide nanoparticles, polymeric nanoparticles, antibiotics, biological extracts, and antimicrobial peptides for controlling infections have been reviewed in detail. Finally, the challenges and future prospects of nanoparticle–hydrogel systems have been discussed.

## Abstract 2

Nanoparticle-hydrogel systems are versatile biomaterials that possess numerous advantages for biomedical applications. This review focuses on the application of the nanoparticle-hydrogels system for the delivery of antimicrobial agents.

## Graphical abstract

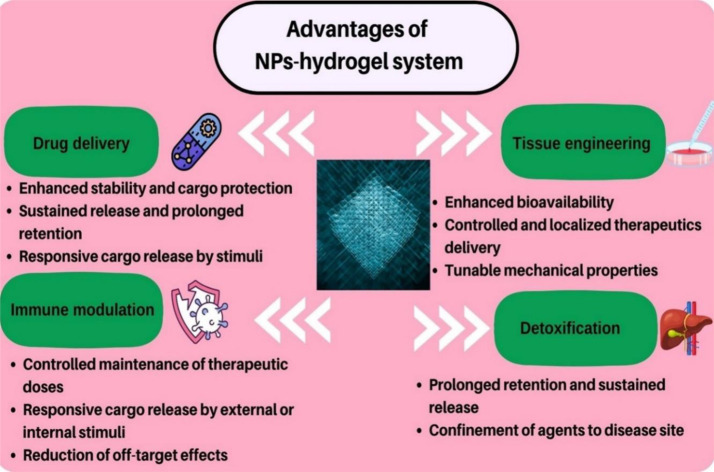

## 1. Introduction

The treatment of local or systematic infections caused by microbes (bacterial viruses, fungi, and parasites) is a major global challenge owing to poor antimicrobial efficacy of conventional antimicrobial agents, the development of antibiotic resistance, and poor patient compliance.^1^ For instance, chronic skin wound infections caused by antibiotic-resistant bacteria have become an imminent global public health concern causing high mortality and a massive economic burden in the healthcare sector.^2,3^ Additionally, despite the rapid development of biomaterials and medical devices, healthcare-associated infections (HAIs) resulted in delayed healing, posing serious problems to clinicians.^4^ According to a World Health Organization (WHO) 2019 report, at least 700 000 deaths worldwide occur every year due to drug-resistant bacteria, and if no action is taken the number of deaths is expected to reach 10 million per year by 2050.^5^ The leading causes of escalating antibiotic resistance associated with various pathogens include misuse or overuse of conventional antibiotics and dwindling pipeline in the development of new antibiotics against resistant microbes.^3,6^ Therefore, there is an imperative need for the development of new strategies to deliver antimicrobial agents to address this challenge.

Commonly, two treatment methods *viz.*, replacement of old materials with new ones and systemic antibiotic therapy are being employed to control antibiotic resistance and HAIs.^2^ However, both of these methods do not provide complete healing and also lead to increased bacterial resistance and mortality along with high cost. Thereafter, exploration and development of different antimicrobial materials, natural extracts with antimicrobial properties, and heavy metals proved to be effective treatments. Despite effectiveness, these materials still have inherent disadvantages that restrict their efficacy when used for treatment. The disadvantages include low efficacy, lack of controlled release, high dose requirement, low bioavailability, short-term effect, toxicity *etc.* To alleviate these issues, there is an urgent requirement for the development of antimicrobial materials with properties such as high efficacy, high bioavailability, ability to provide long-lasting effect, controlled and sustained release capability along with cost-effectiveness.

In this regard, the hydrogels (which represent a class of versatile polymeric biomaterials that can be utilized as prolonged delivery carriers for antimicrobial therapeutics agents) are potential attractive systems for the delivery of the antimicrobial agent. The research on applications of hydrogels in biomedical fields have extensively explored these polymeric systems for engineering tissues, delivering numerous types of therapeutic agents/drugs, healing of wounds, diagnosing diseases, and coating the surface of implants. *etc.*^3,7–9^ Classically, these hydrogels represent a class material that is formed by the crosslinking polymeric chains resulting in porous 3D (mesh-like) network that can uptake water/aqueous fluid due to hydrophilicity and porous structure. The compatibility with cells and tissue, controllable network and pore structure, biodegradability, and ability to change in their structure and properties (in response to internal and external stimuli) renders hydrogel attractive materials in delivery of drugs. Over the last twenty years, hydrogels with antimicrobial effects (due to their constituents) or hydrogels loaded with antibacterial agents, have emerged as attractive candidates to control infections.^3,10^ The advantages offered by hydrogels as antimicrobial delivery vehicles are mainly attributed to their versatile and biocompatible network structure that can be tailored for controlled, sustained, on-demand, and targeted release by changing crosslinking approach or polymer composition.^10^

Despite these advantages of hydrogel, there are some limitations/drawbacks in their application for antimicrobial delivery. For instance, hydrogels fabricated by utilizing natural polymeric are mechanically less stable, load lower amount of drug, have limited ability to control drug release, and are less efficient to protect bioactivity of loaded antimicrobial agents.^11^ Alternatively, hydrogels fabricated from synthetic polymers show network defects which may result in changes in the in bioactivity of the loaded drug compound, rate of drug diffusion, swelling behaviours, and mechanical strength. Therefore, controlled and prolonged delivery of the antimicrobial agents in stable (active) form is a difficult task and further necessitates the exploration of novel strategies to construct advanced and functional hybrid hydrogels by loading various nano/microstructures.^12^ In recent years, to circumvent the issues related to improved drug delivery, hybrid hydrogels incorporating nano/microstructures, peptides or biologically active proteins have been developed with controlled delivery characteristics for tissue engineering, drug delivery, and wound healing applications.^13,14^

In addition, nanoparticle-hydrogel systems (superstructures) also represent an emerging material for overcoming antibiotic resistance and related undesirable side effects by using lower doses of antibacterial agents compared to traditional hydrogels.^14–16^ Over the past several decades, with the advancements in nanotechnology, nanoparticles have been extensively explored for antimicrobial effect (*e.g.* metal nanoparticles) or as carriers for the delivery of drugs with the ability to encapsulate one or multiple therapeutic agents including small molecules, bioactive molecules, antimicrobial agents, peptides, nucleic acids, among others.^17–20^ The advantages of nanoparticle-based drug delivery systems include enhanced drug solubility, improved drug localization and bioavailability for controlled release of loaded therapeutic agents. However, nanoparticles-mediated drug delivery still has some limitations such as premature release of loaded agents, bulk drainage, toxicity in certain organs due to unwanted systemic exposure and cell-mediated transport away from target site.^21–23^ In order to address these pitfalls, nanoparticle-loaded drugs/antimicrobial agents need to be encapsulated within 3D matrices such as scaffolds/hydrogels to improve their stability and targeted drug delivery. In recent years, nanoparticle–hydrogel superstructures have garnered much attention in antimicrobial drug delivery while minimizing drug resistance development owing to their highly tuneable and inherently multifunctional nature.^8,9,13,14,24–26^ In this context, nanoparticles incorporated hydrogels are a promising class of materials for the delivery of antimicrobial agents. It has been well-documented that hydrogels are highly biocompatible and can be used to deliver drugs to specific sites in the body. However, the inclusion of nanoparticles in the design of hydrogel systems offers several additional advantages such as increased stability, enhanced drug release, bestowing stimuli-responsiveness, encapsulating therapeutic agents, enhancing mechanical properties, and improved targeting capabilities.^27–30^ The use of nanoparticles as antimicrobial agents is particularly attractive due to their small size, large surface area, and unique physicochemical properties, which allow for targeted delivery and improved drug bioavailability. In addition, the incorporation of nanoparticles within hydrogels can protect the drug molecules from degradation and increase their stability in biological fluids. Another significant benefit of nanoparticle-incorporated hydrogels is their versatility in delivering a wide range of antimicrobial agents. For instance, antibiotics such as amoxicillin and cephalexin can be incorporated into hydrogels to deliver them directly to the site of infection. This approach reduces the dose of the antibiotic required, minimizing the potential for side effects while increasing the efficacy of the drug.

**Fig. 1:**
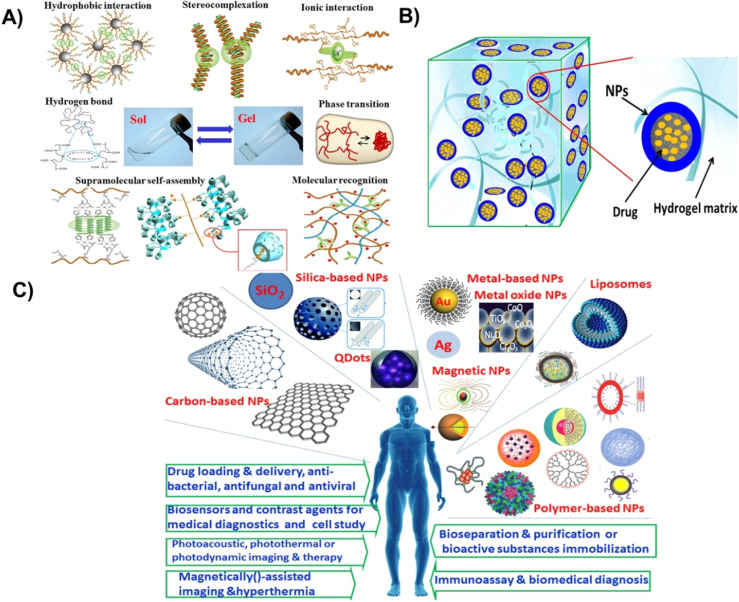
(A) Different methods for fabrication of physically crosslinked hydrogels, (B) hydrogel–nanoparticle hybrid system, (C) biomedical applications of different types of nanoparticles. Reproduced from ref. 35. This work is licensed under a Creative Commons Attribution 4.0 (CC BY) International License.

Significant advancements have been made utilizing nanoparticle–hydrogels systems by combining the advantages of these individual technologies in recent years towards drug delivery, tissue engineering, immune modulation, and detoxification as shown in Fig. 1.^8,14,24^ Moreover, the combined nanoparticle–hydrogel structures modulate drug release kinetics, enhances nanoparticle-based detoxification, assist in localized drug delivery, and functions as tissue engineered matrices. A vast range of nanoparticle–hydrogel combinations have been developed due to the wide availability of hydrogel matrices and nanomaterials for biomedical and pharmaceutical.^26^ Antimicrobial hydrogels that incorporate nanoparticles may be classified into different types: (1) hydrogels incorporating metal nanoparticles with antimicrobial capabilities, (2) hydrogels incorporating polymeric nanoparticles with antimicrobial capabilities, (3) hydrogels incorporating antibiotics loaded nanoparticles, and (4) hydrogels incorporating other antibacterial agents (biological extracts/antimicrobial peptides) loaded nanoparticles.

Recent studies have demonstrated the potential of nanoparticle-incorporated hydrogels for the treatment of various infections such as bacterial and fungal infections.^13,14,25,26^ In earlier research reports, silver nanoparticles incorporated within chitosan-based hydrogels have been shown to exhibit potent antimicrobial activity against various pathogens, including methicillin-resistant *Staphylococcus aureus* (MRSA) and *Candida albicans*.^31,32^ In another study, gold nanoparticles incorporated within alginate-based hydrogels have been reported to possess potent antifungal activity against *Aspergillus fumigatus*.^33^ Furthermore, titanium dioxide nanoparticles have been shown to possess antibacterial properties and can be incorporated into a hydrogel system to inhibit bacterial growth.^34^ Nonetheless, nanoparticle-incorporated hydrogels present an exciting new avenue in the development of advanced drug delivery systems for combating infectious diseases.

This review provides an overview of recent advancements in nanoparticle-incorporated hydrogel systems controlling bacterial infections. An overview of nanoparticle–hydrogel systems, their advantages and applications in drug delivery, tissue engineering, and reducing bacterial resistance has been discussed. Furthermore, we focused mainly on hydrogels encapsulating antimicrobial agents-loaded nanoparticles for efficient antimicrobial delivery. In this context, various antimicrobial agents such as metal/metal oxide nanoparticles, polymeric nanoparticles, antibiotics, biological extracts, and antimicrobial peptides-loaded hydrogels have been discussed in detail with recent advancements. Finally, challenges in clinical transformation and future prospects of these nanoparticle–hydrogel systems have been provided. Taken together, these emerging antimicrobial agents loaded nanoparticle–hydrogel-based systems offer an alternative strategy for antimicrobial delivery in the controlled and sustained manner for overcoming microbial infections and antibiotic resistance.

## 2. Nanoparticle–hydrogel system

Nanoparticle–hydrogel systems represent a relatively new class of biomaterials that are designed to integrate the beneficial characteristics of the individual materials (*i.e.* hydrogels and nanoparticles) to circumvent the drawbacks of either individual nanoparticles or hydrogels for drug delivery, tissue engineering, or other biomedical applications.^36^ In this section an overview of the classification, chemistry, preparation methods, release kinetics, degradation, and injectability of nanoparticle-hydrogel systems for drug delivery is provided.

### 2.1. Classification and chemistry

As discussed above, 3D hydrogel networks that are formed by the cross-linking of the polymer, possess the ability to swell in water/aqueous fluids or other liquids, have versatile applications for the delivery of therapeutics agents, tissue engineering, diagnosis of diseases, and other fields of biomedicines. Based on diversity in their composition, methods of formation, network structure, characteristics, stimuli-responsiveness, and applications, hydrogels are categorized into different classes. For instance, hydrogels are mainly categorized as (i) natural, synthetic, and hybrid based on polymers source used to fabricate hydrogel; (ii) homo-polymeric, co-polymeric, and interpenetrating networks (IPNs) based on polymer composition; (iii) chemical (covalent-interaction), and physical (ionic, hydrogen bonding, hydrophobic, supramolecular interaction) based on the type of interactions; (iv) macro, micro, nano, bead, films, matrix, injectable-gels, *etc.* based on the size and physical form of application; (v) cationic, anionic, zwitterionic, non-ionic, and amphoteric based on charge on hydrogel network; (vi) physical stimuli (*e.g.* temperature, electrical, photo), chemical stimuli (*e.g.* pH, ionic strength/salt), biological stimuli (*e.g.* antigen, glucose, enzyme responsive) responsive based on the stimuli-responsiveness; (vii) crystalline and amorphous based on the crystallinity of network and; (vii) degradable and non-degradable based on the degradability of network.^37–39^ Additionally, hydrogels may also be categorized and sub-categorized into different classes depending on particular character and application fields.

Nanoparticles represent a large class of materials that deal with particles in the size ranges of manometer (10–1000 nm) possessing versatile properties and applications across the whole spectrum of pharmaceutical and biomedical science. The nanoparticles can be categorized into various categories based on their source, chemistry, dimensions, characteristics, and applications.^40,41^ However, nanoparticles are mainly classified as organic (polymer, lipid, DNA, protein, *etc.*), and inorganic nanoparticles (metallic, metal oxides, ceramic, *etc.*).^41,42^

In literature, various terms have been used for nanoparticle hydrogel systems that comprise nanoparticles incorporated into the hydrogel systems. The most commonly used terms include nanocomposite/composite hydrogels, hybrid hydrogels, and nanoparticle-hydrogel superstructures.^14,25,36,37,43,44^ All these terms mainly represent the hydrogel systems that contain nanoparticles in their three-dimensional network structure either crosslinked or uncrosslinked.^44^ Therefore, while describing nanoparticle hydrogel systems (in this review) the term “hybrid/hybrid systems” should not be mixed with “hybrid hydrogel” that are the hydrogels fabricated by utilizing the combination of polymers from different sources (natural and synthetic polymers).^37^ Moreover, these nanoparticle-hydrogels systems are called nanocomposite/composite hydrogels because they contain two different types (nanoparticles and hydrogels) of materials (having unique and different characteristics) that a combined in such a way that they maintain their identity while showing distinct different material characteristics that help to overcome the drawbacks of individual materials.^35,36^

The nanoparticles–hydrogel systems can also be divided into different classes like hydrogels or nanoparticles on the basis of the polymers source (natural, synthetic), the chemistry of synthesis (physical and chemical), method of preparation, stimuli-responsiveness (pH, ionic strength, enzymes, thermal *etc.*), type of nanoparticle incorporated (metal, polymeric, liposome, micelle, metal oxide nanoparticles incorporate) and biomedical/pharmaceutical application. In this review, we summarize nanoparticle–hydrogels system for antimicrobial effect based on the type of antimicrobial nanoparticle incorporated into hydrogels *i.e.* (i) hydrogels incorporating metal nanoparticles with antimicrobial capabilities, (ii) hydrogels incorporating polymeric nanoparticles with antimicrobial capabilities, (iii) hydrogels incorporating antibiotics loaded nanoparticles, and (iv) hydrogels incorporating other antibacterial agents (biological extracts/antimicrobial peptides) loaded nanoparticles.

With regard to chemistry, the nanoparticle-hydrogel systems can be synthesized by physical and chemical interactions.^14,37^ In the case of physically synthesized nanoparticles–hydrogel systems, the non-convent interactions (like ionic-bond, hydrogen-bond, coordination bond, hydrophobic interaction, supramolecular interaction, stereo–complexation interaction, *etc.*) are employed to: (i) crosslink the polymeric chains with each other to form hydrogels network in which nanoparticles can be incorporated later; (ii) crosslink nanoparticles and polymeric chains to form as nanoparticle-hydrogel network; or (iii) for the interactions between the nanoparticles the behave like a nano-gel.^14,36,37,43^ Since these physically interacted nanoparticle-hydrogel systems do not require the use of potentially toxic chemicals (crosslinkers, initiators, chemicals), therefore, these systems are more biocompatible and cost-effective than chemically interacted systems.^14^ In addition, the physically interacted nanoparticle-hydrogels are attractive options to impart stimuli responsiveness thereby fine-tuning the release of the incorporated nanoparticles or drug at the targeted site using suitable stimuli.^14,37^ Despite these advantages the major drawback of these systems is their weak mechanical properties and instability due to weak reversible nature of these physical interactions^14,37^ On the other hand, hydrogel networks can also be obtained by the chemical crosslinking (covalent bonding) of the polymers using free-radical, click chemistry, enzymatic, Michael-type addition, radiation-induced, and Schiff bases polymerization reactions.^14,37^ These covalently crosslinked hydrogels have stronger networks compared to physical interaction thereby providing better mechanical characteristics and stability. The degree of crosslinking of these hydrogels can be tuned to control the degradability and degradation of the hydrogels. Moreover, these chemically crosslinked hydrogels can also be designed to impart them stimuli-responsiveness target released of the incorporated nanoparticles and other incorporated therapeutic agents.^14^ However, due to the use of the solvent, initiators, and crosslinkers in the synthesis reaction the chemically crosslinked hydrogels may be toxic, therefore, attention should be paid in the selection of these reagents and purification of the hydrogels.^36,43^ In literature, both physically and chemically interacted systems have been reported for the delivery of various therapeutic agents and other biomedical applications.

### 2.2. Preparation methods

The design of the homogeneous nanoparticle–hydrogel system is a challenging task that requires the careful selection of the appropriate approach/method depending on the nature of the nanoparticles and hydrogels as well as the intended use of the nanoparticle–hydrogel systems. In literature, five major methods/approaches have been reported to prepare nanoparticle–hydrogel systems that include: (i) fabrication of hydrogels in a suspension of nanoparticles; (ii) entrapment of the nanoparticles into pre-formed hydrogel networks; (iii) *in situ* fabrication of the nanoparticle inside pre-formed hydrogel networks, (iv) fabrication of hydrogels employing nanoparticles for crosslinking, and (v) preparation of the preparation of hydrogel coated nanoparticles. These approaches/methods for the preparation of the hydrogel have been discussed in detail in previous review articles.^1,26,34,36,45^ However, a brief summary of these methods for the preparation of the nanoparticles–hydrogels systems is provided in Table 1 along with advantages, drawbacks, and some examples of each method.

**Table 1:** Summary, advantages, and drawbacks of methods for the preparation of the nanoparticles-hydrogels systems.^1,26,34,36,45^

Preparation method	Brief description	Advantages	Drawbacks	Examples
Fabrication of hydrogels in a suspension of nanoparticles	Fabrication of hydrogel network by adding monomers, crosslinker and initiator (required to fabricate hydrogel networks) in suspension of nanoparticles	Allows incorporation of various types of nanoparticles into hydrogels networks, simple, and widely used	Possibility of nanoparticle aggregation in hydrogel, nanoparticle interaction with hydrogel/gelators, nanoparticle leaking from loosely crosslinked hydrogels, and use of potentially toxic crosslinkers that require extensive/expensive purification process	Fabrication of *N*-isopropylacrylamide/acrylamide hydrogel in gold nanoparticle suspension,^46^ fabrication of hydrogels based on acrylate in silver nanoparticles suspension^47^
Entrapment of the nanoparticles into pre-formed hydrogel networks	Nanoparticles are physically entrapped into hydrogel networks by repeatedly swelling and deswelling preformed hydrogels into nanoparticle dispersion (swelling medium) and shrinking medium, or nanoparticles are physically incorporated in hydrogel by cyclic centrifugation, heating or re-dispersion	Can reduce drawbacks of nanoparticle aggregation, and eliminate possibility of chemical interactions between nanoparticles and hydrogel/gelators	Leakage of the nanoparticle from hydrogel due to weak physical interaction, achieving efficient entrapment of nanoparticles, and use of organic solvent for deswelling are major limitations	Swelling and deswelling of acrylamide-based hydrogels in aqueous gold (Au) nanoparticle dispersion (swelling) and organic (shrinking) media repeatedly for Au nanoparticle-containing hydrogels^48^
				Placing dry chitosan-based hydrogel in an aqueous silver (Ag) nanoparticle dispersion to physically entrap Ag nanoparticles into hydrogel network^49^
*In situ* fabrication of the nanoparticle inside pre-formed hydrogel networks	Metal ions (precursor of nanoparticles) are introduced into hydrogel network where *in situ* fabrication of the nanoparticles are by reduction process adding reducing agents or light	Simple, minimum aggregation of nanoparticles, ease of scalability, and incorporation of large number of nanoparticles	Limited mainly to nanoparticles of metals and metal oxide	Silver and gold nanoparticles were prepared inside PNIPAM or PAA hydrogels using a reducing agent (sodium borohydride).^50^
				Zinc oxide nanoparticle formation inside carboxymethyl chitosan (CMCs) hydrogel using zinc nitrate and reducing agent^51^
Fabrication of hydrogels employing nanoparticles for crosslinking	The functional groups present on the nanoparticles are utilized as crosslinker to fabricate hydrogel	The ability to form multiple bonds (multivalency) in hydrogel network, enhanced mechanical stability and flexibility	May limit the ratio of nanoparticles to form stable nanoparticle-hydrogels due to co-dependency of nanoparticles and hydrogel	Fabrication PNIPAAm hydrogel using vinyl functionalized gold nanoparticles by copolymerization^45^
Preparation of hydrogel coated nanoparticles	The nanoparticles are added to the polymers solution along with crosslinker and other reactants to obtain hydrogel coated nanoparticles	Reduced aggregation of nanoparticles, possibility of loading wide variety of therapeutic agents, protection of loaded agent, and ease of scalability	Complicated procedure of preparing and purification that requires more time. Possibility of toxicity due to residual reactants	Polyaniline-coated silica nanoparticles hydrogel^52^
				Vancomycin-loaded silica xerogel core-shell hydrogel prepared by double emulsion method^53^

### 2.3. Release mechanism

One of the main objectives of incorporating nanoparticles into hydrogel systems is to improve the stability and release properties of the incorporated nanoparticles or therapeutic agents. The release of entrapped drugs or other agents from the hydrogel networks generally occurs by three main mechanisms that include: (i) diffusion of the entrapped molecule, (ii) swelling of the hydrogel network, and (iii) chemically controlled that mainly involve the degradation/erosion of the hydrogel network.^25,54,55^ The loading method, the composition of constituent polymers, network structure, and physiochemical characteristics play key roles in determining the release mechanism from hydrogels, therefore, hydrogels can be tailored for controlled release by modulating these factors.^54^ In literature, numerous mathematical models have been proposed to estimate release kinetics from hydrogels.^54,55^ However, due to the hydrophilicity of the network, the hydrogels are more efficient for the delivery of water-soluble molecules.^56^ The burst release of payload owing to quick diffusion from some hydrogel networks also represents another limitation of hydrogel delivery systems.^25^

On the other hand, two major mechanisms have been reported to control the release of therapeutic agents from nanoparticles *i.e.* by linker cleavage (for agents linked through covalent bonds) and carrier control (for agents loaded *via* non-covalent interaction or physical entrapment) as comprehensively described in previous reviews.^57,58^ Therefore, the release kinetics from nanomaterial can be fine-tuned based on the nature of the loaded agents as well as the target organ by varying the composition, preparation strategy, size, charge, and shape.^14,25^ Nanoparticles can be designed to be stimuli-responsive which can be utilized to target the release of payload at specific sites in the body.^14^ Thus, the incorporation of the nanoparticles in the hydrogels can combine the inherent beneficial characteristics of these two delivery systems and provide an opportunity to more efficiently modulate the release characteristics by overcoming the limitation of individual systems.^14,25^ These versatile nanoparticle-hydrogel systems can overcome numerous delivery challenges that improve compliance of patients like overcoming the burst release by prolonging sustained release, delivering a wide variety of molecules, improving the stability of nanoparticle/drugs, and reducing toxic/side effects by releasing payload at the target site.^14,25^ However, general mechanisms are possible including (i) incorporated nanoparticles are first slowly released from hydrogels (by mechanism discussed above) and then drug encapsulated into nanoparticle is released (*via* mechanism discussed above); (ii) release of drug from nanoparticles inside the hydrogels network which is then released from hydrogel network (iii) combination of (i) and (ii).^56^ The predominant mechanism governing the release from a particular nanoparticle–hydrogel system may depend upon the characteristics and composition of the individual nanoparticles and hydrogel (as discussed above) as well as the preparation approach and nature of the drug. Overall, the release of the incorporated nanoparticles or nanoparticles loaded with therapeutic agents from the hydrogel networks can be tailored by careful selection of the hydrogel (polymer composition, preparation approach, crosslinking, pore size, stimuli-responsiveness function, *etc.*) nanoparticles (composition, linker, size, shape, properties, stimuli-responsiveness), preparation method for incorporation of nanoparticle into hydrogel system and desired biomedical/pharmaceutical application.

### 2.4. Injectability

The injectable hydrogels, which behave as liquid/fluid (sol) when extruded from the syringe/needle but are converted to soft solid (gel) after extrusion/injected inside the body, can provide numerous benefits for clinical applications including less invasiveness, do not need open surgery for implantation, lesser post-implantation complications, minimizing side effects, reduce pain, short recovery time, stimuli-responsiveness, low cost and improving patient compliance.^59,60^ These hydrogels can be divided into two main types based on the injectability mechanism namely *in situ* and shear-thinning injectable gels. In the first type (*in situ* gelling) a sol of the precursor of hydrogel gel (low viscosity) is injected that transforms into gel after injection (implementation) owing to *in situ* crosslinking (physical or chemical).^60^ In the second type of injectable hydrogels (shear-thinning), firstly a gel is formed outside the body with shear-thinning characteristic (*i.e.* their viscosity is decreased upon application of shear stress) and injected to the desired site of application where it again transforms into a gel upon removal of shear force (also termed as self-healing of gel).^60^ Despite the advantages, injectable hydrogels (alone) fail to achieve the desired mechanical and other physiochemical characteristics for biomedical applications that can be overcome by enforcing/adding other materials (fillers or nanoparticles).^61^

The combination of nanoparticles and hydrogels combines or synergies the characteristics of individual materials in single injectable systems.^8^ In addition to improving the mechanical and other physiochemical characteristics of the injectable hydrogel (alone), the incorporation of the nanoparticles into injectable hydrogels provides numerous benefits such as the delivery of hydrophobic drugs, sustained and targeted release, promote cell attachment, and proliferation.^8^ Therefore, numerous nanoparticle-incorporated injectable hydrogels have been prepared (using different nanoparticles and polymers) and explored for drug delivery, preventing infection, tissue engineering, wound healing, and other biomedical applications (as reviewed previously).^60,61^ Some examples of the antimicrobial injectable nanoparticle–hydrogel systems in this review (under relevant Sub-Sections).

### 2.5. Biodegradation and fate

The degradability of biomaterials is an important characteristic of their pharmaceutical and other biomedical applications. Therefore, the degradability of the nanoparticle-hydrogel systems should be considered while designing such systems for drug delivery, wound healing, tissue engineering, diagnostics, and other biomedical applications. Concerning hydrogel-based biomaterials for drug delivery applications the biodegradability of such systems provides the ease of removing such systems after the release of the incorporated therapeutic agent or nanomaterial. However, while designing such hydrogels-based systems a balance should be maintained between degradability and the mechanical stability of the hydrogel which can be built in the hydrogels by careful selection of the polymers based on chemistry, degradation conditions, and intended site of application. The premature degradation of the hydrogel may limit the desired therapeutic effect and non-degradability may result in toxic or immunogenic effects. Numerous degradation mechanisms of the polymeric networks have been reported including: (i) solubilization of polymers; (ii) mechanical degradation; (iii) physical bond degradation; (iv) chemical degradation; (v) photo-degradation; and (vi) hydrolytic degradation.^62,63^ However, this classification of the degradation mechanism can be different based on the polymers used in the hydrogel fabrication and intended application. In some hydrogels, a combination of the degradation mechanisms may be involved in the complete degradation of the 3D network and individual polymers. The details of the degradation mechanism of the hydrogels are beyond the scope of this review, however, these mechanisms have been comprehensively reviewed in some recent articles.^62,63^

The prediction and study of *in vivo* (especially in the human body) fate of the nanoparticles (organic or inorganic) after release from the nanoparticle-hydrogels systems is complex and cannot be generalized. Numerous factors play a role in determining the absorption, distribution, degradation, metabolism, distribution, deposition (accumulation), clearance, and toxicity of the nanoparticles from the body including type (polymeric/inorganic), source (natural synthetic), chemical structure, biodegradability, physiochemical characters (size, shape, charge, *etc.*), stimuli responsiveness, and concentration of the nanoparticles.^64,65^ In addition, the fate of the nanoparticles also depends upon the route of administration *i.e.* topical, parenteral, ocular, inhalation, and oral, *etc.*^65^ The details of the absorption distribution, metabolism, and elimination (ADME) of the different types of nanoparticles administered through different routes have been described in detail by the researcher.^64,66^ Therefore, while developing nanoparticle–hydrogel systems for pharmaceutical and biomedical applications detailed investigation of their *in vivo* fate of the system should be conducted.

## 3. Advantages of NPs incorporated hydrogels in delivery of the antimicrobial agents

**Fig. 2:**
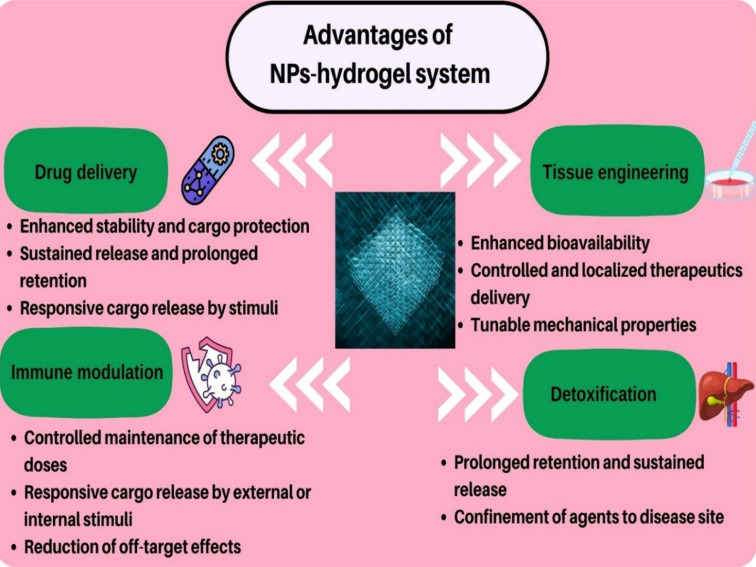
Applications and advantages of nanoparticles (NPs)–hydrogel systems in different fields of biomedical science.

Nanoparticle–hydrogel hybrid systems can be employed for various biomedical applications including tissue engineering, drug delivery, immune modulation, reducing antibacterial resistance, detoxification, and wound healing owing to their unique advantages (Fig. 2).

The most notable advantages of nanoparticle–hydrogel platforms include enhanced drug delivery properties. Other advantages of this hybrid platform in antimicrobial therapy include:

• Increased efficacy: incorporating nanoparticle-based antimicrobial agents in hydrogels increases their efficacy as the nanoparticles offer a high surface area for interaction with microbes.

• Controlled release: nanoparticles incorporated hydrogels offer a controlled release mechanism for antimicrobial agents, which increases their effectiveness and reduces toxicity.

• Long-lasting: the nanoparticles incorporated in hydrogels offer long-lasting efficacy for the antimicrobial agents, which reduces the need for frequent applications.

• Enhanced bioavailability: the nanoparticles offer enhanced bioavailability by increasing the solubility, absorption, and distribution of the antimicrobial agents.

• Targeted delivery: nanoparticles incorporated hydrogels allow for targeted delivery of the antimicrobial agents, reducing potential harm to healthy cells and tissues.

• Low toxicity: the use of nanoparticles and hydrogels helps reduce the toxicity of the antimicrobial agents, reducing the side effects and adverse reactions in patients.

• Biocompatible: nanoparticles incorporated hydrogels are biocompatible and can be used for both topical and systemic delivery of antimicrobial agents.

• Improved wound healing: the incorporation of nanoparticles and hydrogels can improve wound healing by reducing the risk of infections and promoting tissue regeneration.

• Cost-effective: nanoparticles incorporated hydrogels offer a cost-effective alternative for the delivery of antimicrobial agents, reducing the need for multiple applications and lowering the cost of treatment.

Moreover, the hybrid NPs-hydrogels material can offer a numerous characters for different applications in biological systems for example, tuned cellular response, improved mechanical and physical properties, stimuli-responsive material behaviour, modulation of drug release kinetics and drug release capability in controlled and “on-demand” fashion along with site-specific drug targeting.^14^ Nonetheless, both the hydrogel and nanoparticle components in this hybrid platform can be modulated to improve the bioavailability of the loaded agents at the delivery site in controlled manner without systemic toxicity when administered into the body.

### 3.1. Drug delivery

Drug delivery represents a process to deliver a pharmaceutical agent to achieve desired therapeutic effects in the patient's body. Over the last two decades, both hydrogel and nanoparticle-based systems have been extensively explored separately for drug delivery applications.^67,68^ Nanoparticle-based delivery alters the pharmacokinetic and biodistribution profiles of therapeutic agents and helps in improving the therapeutic efficacy of drugs. Many nano-formulations have been successfully translated for clinical applications.^20,69–71^ On the other hand, owing to their unique properties such as porous structure, water-absorbing ability, and stimuli-responsive nature, hydrogels have been also leveraged for drug delivery applications.^72,73^ Currently, with recent developments in material design and emerging technologies, nanoparticle–hydrogel systems have been developed and utilized for improved drug delivery of therapeutic agents.^25^ The concept of hybrid material-based delivery platform centres around the creation of a versatile hierarchical delivery system with multiple components (nanoparticles, hydrogels, and therapeutic agents), which was otherwise not possible to achieve with either nanoparticles or hydrogels independently. Moreover, nanoparticle-hydrogel platforms have emerged as a promising strategy in drug delivery due to their unique properties that allow for sustained release of drugs and efficient targeting of specific tissues or cells. Nanoparticles, which are particles with a size between 1 and 100 nm, can be designed to encapsulate drugs and protect them from degradation or elimination prior to reaching their target site. Hydrogels are three-dimensional networks of crosslinked polymer chains that can absorb large amounts of water without dissolving, giving them unique properties including high swelling and elasticity. They can be engineered to release drugs over a sustained period of time, enabling controlled and targeted drug delivery. The combination of nanoparticles and hydrogels can enhance drug delivery beyond what either system could achieve alone. Nanoparticles can be embedded within a hydrogel matrix to improve the stability of the drug and provide a slower, sustained release. Additionally, the hydrogel matrix can act as a protective barrier against degradation and clearance of the nanoparticles, extending their circulation time in the body. Moreover, the properties of nanoparticle–hydrogel platforms can be tuned to achieve targeted drug delivery. The nanoparticles can be functionalized with targeting ligands that recognize specific receptors on the surface of cells or tissues, while the hydrogel can provide a physical barrier that prevents nonspecific binding and uptake of the drug by healthy cells. Furthermore, nanoparticle–hydrogel composites can be tailored to exhibit environmental responsiveness, meaning they can release the drug in response to specific stimuli, such as changes in temperature, pH, or concentration of biomolecules. This enables targeted delivery of the drug to diseased or damaged tissue sites, further enhancing therapeutic efficacy.

Nanoparticles incorporated hydrogels have several advantages in the delivery of antimicrobial agents in drug delivery, including:

• Controlled release: nanoparticles-incorporated hydrogels can release the antimicrobial agents in a controlled manner, ensuring a sustained therapeutic effect over a longer period of time.

• Increased effectiveness: nanoparticles in the hydrogel systems increase the effectiveness of the antimicrobial agents by penetrating the bacterial biofilm and evading bacterial resistance, resulting in higher antimicrobial activity.

• Enhanced stability: nanoparticles can protect the antimicrobial agents from degradation and improve their stability, allowing for longer shelf life and increased effectiveness.

• Reduced toxicity: antimicrobial agents incorporated in nanoparticles have reduced toxicity compared to free antimicrobial agents and minimize the risk of cytotoxicity to the host cells.

• Targeted delivery: nanoparticles-incorporated hydrogels can be designed to target specific tissues and organs, ensuring the antimicrobial agents are delivered precisely to the site of infection, thus increasing their effectiveness.

• Non-invasive: nanoparticles-incorporated hydrogel systems can be administered non-invasively, reducing patient discomfort and the risk of infection associated with invasive procedures.

• Cost-effective: nanoparticles-incorporated hydrogels are cost-effective and can be easily manufactured using standard techniques, making them more accessible to a larger section of the population who may require antimicrobial therapy.

• Improved bioavailability: nanoparticles can improve the bioavailability of poorly soluble drugs by enhancing their solubility and absorption.

• Combination therapy: nanoparticle-hydrogel superstructures can be used to deliver multiple drugs simultaneously, allowing for combination therapy and improved therapeutic outcomes.

Nanoparticle encapsulation within hydrogel structures further enhances the stability of encapsulated drugs, as well as prevents their premature release. In earlier studies, porous silicon nanoparticles have been utilized as biocompatible drug carriers with high loading capacities.^74,75^ However, fast, and uncontrollable drug release limits the utility of the nanoparticle-based delivery platform. In order to address these pitfalls, porous silicon nanoparticles were first conjugated with gold nanorods followed by encapsulation within a calcium-alginate hydrogel structure to enable the controlled release characteristics along with prevention of drug leakage.^76^ In another study, drug-loaded metal–organic framework (MOF) nanoparticles were encapsulated into acrylamide/DNA hydrogels to enhance drug loading efficiency and reduce model drug leakage as compared to duplex DNA-capped MOF nanoparticles.^22^

Hydrogels protect the encapsulated biological compounds from degradation and elimination along with promotion of bioavailability enhancement. Various research groups demonstrated enhanced stability and activity of the nanoparticles loaded with small interfering RNA (siRNA) and microRNA incorporated within hydrogels through modulation of release kinetics and degradation rate.^23,77^ Nanoparticle-hydrogel based platforms have also demonstrated inhibition of bacterial growth without affecting the mammalian cell viability with sustained release of silver for two weeks.^24^ In another study, an adhesive nanoparticle–hydrogel hybrid system was developed for localized antimicrobial drug delivery.^78^ In this hybrid delivery system, mussel-inspired catechol-based hydrogel incorporated an antibiotic, ciprofloxacin loaded poly (lactic-*co*-glycolic acid) (PLGA) nanoparticles for controlled and sustained release to inhibit formation of bacterial biofilms without being structurally damaged.^79^

Nanoparticles of metals such as silver and gold possess various unique physicochemical, electronic, and optical properties such as surface plasmon resonance.^80,81^ In photothermal therapy for cancer, nanoparticles with light absorption property have been often employed. In this therapy, heat generated *via* irradiation induces hyperthermia for cancer therapy.^82,83^ In an earlier study, anticancer drug loaded polydopamine (PDA) nanoparticles incorporated within hydrogels have demonstrated effective for tumour ablation owing to their photothermal effect and high loading capabilities.^84^ PDA nanoparticles released the drug an on-demand manner with near-infrared (NIR) irradiation *via* π–π stacking. There was minimum drug release from hydrogels without irradiation, suggestive of the effectiveness of NIR-responsive nanoparticle-hydrogel hybrid platform. In recent years, several reports demonstrated utilization of near-infrared (NIR)-responsive nanoparticle–hydrogel systems to control the tumour growth in *in vivo* conditions.^76,84,85^ The nanoparticle–hydrogel hybrid delivery platform further boosts the therapeutic strategy for cancer therapy by providing high dose of the drug to tumours. Chen *et al.*, utilized α-cyclodextrin-functionalized hyaluronic acid hydrogels incorporated with drug-loaded mesoporous silica nanoparticles for effective and targeted delivery to tumours.^86^

Targeted drug delivery is highly useful in biomedical applications to minimize the off-target effects. The site-specific delivery employs biological and chemical stimuli to trigger specific drug release.^87^ In nanoparticle–hydrogel hybrid systems, nanoparticles help in enabling more complex and tunable drug release kinetics, while stimuli-responsive hydrogels provide on-demand drug delivery. In an earlier report, pH-responsive chitosan/alginate hydrogels incorporating nanoparticles loaded with an anti-inflammatory tripeptide demonstrated degradation in the colon to treat inflammatory bowel disease with minimum weight loss and reduced intestinal inflammation.^88^ The pH responsive nanoparticle–hydrogel delivery platform breaks down in intestinal fluid to release the anti-inflammatory tripeptide for treatment of colitis in mouse model. In case of colon cancer treatment, limited efficacy and lack of specificity chemotherapeutic drugs limits the treatment.^89^ To address the shortcomings of oral administration of chemotherapeutic drugs, a multifunctional hybrid system based on chitosan/alginate hydrogels based was developed.^90^ Herein, fab′-functionalized nanoparticles targeting CD98 were co-loaded with a chemotherapeutic drug and siRNA within hydrogel for their upregulated release during progression of human colon cancer. The hybrid platform exhibited pH-responsive property protected the drug-loaded nanoparticles transport and assisted site-specific delivery to the colonic lumen after oral administration.

The application of nanoparticle–hydrogel drug delivery systems has also paved the way for ophthalmic delivery such as glaucoma therapy. Commonly utilized eye drops-based treatment for glaucoma suffers from various limitations such as low bioavailability and short duration of activity due to precorneal tear clearance and the permit-selective corneal epithelium.^91^ In order to alleviate these limitations, there is pertinent requirement for treatment employing delivery system that can release drugs for extended period of time to reduce dosing frequency.^92^ Yang *et al.*, fabricated a hybrid dendrimer hydrogel/PLGA nanoparticle platform to maintain significantly higher concentrations of medicine in the aqueous humour and cornea in glaucoma treatment than PLGA nanoparticles alone.^93^ Overall, nanoparticle–hydrogel systems offer a versatile platform and have shown promise for a wide range of applications in various areas of drug delivery.

### 3.2. Tissue engineering

Tissue engineering is the field of biomedical engineering that aims to create functional tissues and organs in the laboratory through the combination of living cells, scaffolds, and biologically active molecules.^94^ It involves the utilization of biological systems and engineering principles to create artificial living tissues that can be implanted into the body to repair or replace damaged tissues and organs. Some of the applications of tissue engineering include the development of skin substitutes, cartilage repair, bone regeneration, and even the generation of functional organs such as the heart or liver.^8,9,95,96^ Hydrogels have been extensively used in tissue engineering due to their ability to mimic the extracellular matrix (ECM) in living tissues. Hydrogel-based scaffolds can provide a supportive framework for cells to adhere, proliferate, and differentiate, ultimately leading to tissue regeneration.^3^ Various techniques such as 3D printing, electrospinning, and micro moulding can be used to fabricate hydrogel-based scaffolds with complex geometries and controlled architecture.^8,9,97,98^ Hydrogels offer several advantages over traditional tissue engineering materials. They can be tailored to mimic the natural environment of different tissues in terms of mechanical, chemical, and biological properties. They can also be customized to deliver different bioactive molecules, such as growth factors, cytokines, and drugs, to support tissue regeneration. Different types of hydrogels, such as natural and synthetic hydrogels, have been used in tissue engineering to mimic various types of tissues, including cartilage, bone, skin, and nerve tissue.^8,9^

In recent years, nanoparticle–hydrogel systems have been extensively utilized in tissue engineering owing to their unique properties such as ability to mimic the extracellular matrix, tuneable porosity, improved mechanical strength, controlled drug release, tailored biological performance, and electrical conductivity.^8,9,99,100^ In addition, nanoparticle–hydrogel platforms can also be used to deliver therapeutic agents locally to the site of tissue regeneration. By modifying the surface of nanoparticles, they can be functionalized to target specific cells or tissues, improving the efficiency of drug delivery. The properties of developed nanohydrogel composites are directly affected by the combination of nanomaterials and hydrogel. Therefore, while fabricating nano-composite hydrogels, there should be strict control over the dispersion degree of nanomaterials within hydrogel and its mutual combination with the molecular chains of the hydrogel. Nanoparticle-incorporated hydrogels have several advantages in the delivery of antimicrobial agents in tissue engineering. Some of these advantages include:

• Controlled release: nanoparticle-incorporated hydrogels can provide controlled release of antimicrobial agents over a long period of time. This ensures continuous and sustained delivery of the antimicrobial agent to the targeted area, which is essential for effective treatment.

• Enhanced stability: incorporation of nanoparticles into the hydrogel matrix can improve the stability of antimicrobial agents, protecting them from degradation or inactivation in the body.

• Increased bioavailability: nanoparticle-incorporated hydrogels can increase the bioavailability of antimicrobial agents. The nanoparticles can penetrate into the tissue and deliver the antimicrobial agent close to bacteria that may be difficult to reach.

• Targeted delivery: nanoparticle-incorporated hydrogels can provide targeted delivery of the antimicrobial agent to the specific site of infection, reducing the chances of side effects and toxicity to healthy tissue.

• Compatibility with tissue engineering scaffolds: nanoparticle-incorporated hydrogels are highly compatible with tissue engineering scaffolds, ensuring optimal growth and regeneration of the host tissue.

• Antimicrobial efficacy: nanoparticle-incorporated hydrogels have been shown to have superior antimicrobial efficacy compared to conventional drug delivery systems, due to their ability to deliver the antimicrobial agent in a sustained and controlled manner.

• Enhanced cellular Interaction: with nanoparticle–hydrogel systems in tissue engineering, the nanoparticles can act as carriers of growth factors and cytokines which facilitate better cellular interaction with the hydrogel matrix. This facilitates enhanced proliferation and differentiation of cells which leads to better tissue regeneration and repair.

• Biocompatibility: nanoparticle–hydrogel systems are biocompatible and can be customized to suit the needs of the specific tissue being regenerated. They not only facilitate cell adhesion and proliferation but also promote angiogenesis, which leads to better vascularization of the tissue.

• Controlled release of bioactive agents: nanoparticles act as nano-carriers that can be loaded with bioactive agents such as drugs, growth factors, and cytokines. The release of bioactive agents can be precisely controlled by the nanoparticle–hydrogel system, which can lead to better therapeutic outcomes.

• Targeted cell delivery: In tissue engineering, it is essential to deliver cells to specific sites where tissue regeneration is required. The nanoparticle-hydrogel system facilitates targeted cell delivery, which improves the efficacy of the tissue regeneration process.

• Improved retention of cells and bioactive agents: the hydrogel matrix in the nanoparticle–hydrogel system provides a stable and supportive environment that improves the retention of cells and bioactive agents. This ensures that the cells and bioactive agents stay in the targeted site for a longer time, allowing for better tissue regeneration.

• Versatility: the nanoparticle–hydrogel systems can be customized to fit the needs of different tissue types. This means that it can be used in a range of tissue engineering applications, including bone regeneration, cartilage repair, wound healing, and organ engineering.

Nanoparticles, such as gold nanoparticles, iron oxide nanoparticles, and silica nanoparticles, can be incorporated into hydrogels to enhance their physical, mechanical, and biological properties.^9^ The use of nanoparticles in hydrogels can also boost their therapeutic efficacy by allowing for controlled drug or gene delivery. Nanoparticle–hydrogel composites can be used to engineer various tissues, including bone, cartilage, skin, and neural tissues.^8,9,95,96^

The incorporation of nanoparticles in hydrogels has also been shown to enhance the mechanical properties of the resulting composites, making them suitable for load-bearing tissue engineering applications. One application of the use of nanoparticle–hydrogel in tissue engineering is in the development of scaffold materials for bone tissue engineering. Heo *et al.*, developed nanoparticle–hydrogel system using gold nanoparticles and gelatin hydrogels for bone regeneration.^101^ The hybrid gold-nanoparticle–hydrogel composites released gold nanoparticles in a sustained manner and demonstrated improved osteogenic differentiation of mesenchymal stem cells (MSCs) when placed into parietal bone defects in rabbits. Calcium phosphate nanoparticles can be incorporated into hydrogels to create a composite material that mimics the composition of natural bone.^102^ The addition of nanoparticles enhances the mechanical properties of hydrogel and promotes osteogenic differentiation of stem cells. Hu *et al.* reported the development of nanocomposite hydrogel with improved mechanical properties using polyacrylamide/nano-hydroxyapatite (nHAp) for better adhesion of mesenchymal stem cells (MSCs) and osteogenic differentiation promotion.^103^ In another study, Jeong *et al.* also reported significantly improved mechanical strength, cell adhesion function, and osteogenic differentiation of MSCs using nHAp incorporated heat-responsive poly (ethylene glycol)-poly(l-alanine-*co*-l-phenylalanine) (PEG-PAF)/calcium phosphate hydrogels.^104^ In addition to its role in promoting osteogenesis, nHAp incorporation in hydrogels is also reported to regulate the bone immune micro-environment and enhancement of vascular endothelial cell activity.^95,96,105^

Another application of the innovative combination of NPs and hydrogels lies in the engineering of soft tissues such as cartilage, thereby enhancing the cartilage repair capacity.^8,106,107^ In an earlier report, silica nanoparticle–hydrogel composites have been utilized to engineer cartilage tissue and enhance the chondrogenic differentiation of MSCs.^8^ Furthermore, Zhu and coworkers developed hydrogels for cartilage repair using nHA and polylactic acid along with bone marrow derived MSCs to repair rabbit full-thickness cartilage defects. Enhanced chondrogenesis and effective cartilage repair were revealed through histological and immunohistochemical analyses along with enhanced accumulation of extracellular matrix.^108^ In another study, nanocomposite hydrogels with multi-walled carbon nanotubes demonstrated enhanced expression of core-binding factor alpha-1 (CBFA1) and collagen type I alpha-1 (COLIA1), suggesting improved chondrogenesis and regulation of human adipose-derived stem cells responses.^109^ With the recent development in 3D printing technology, researchers have developed multilayer hydrogels incorporating graphene oxide and hyaluronic acid nanoparticles with gradient hardness to effectively repair cartilage defects.^110,111^ The application of nanoparticle–hydrogel hybrid has been extended to neural tissue engineering with the emergence of 3D printing technologies for ready fabrication of scaffolds matching defect sites using functional biomaterials. Tao *et al.*, developed nanoparticle-embedded nerve conduits using digital light processing (DLP)-based 3D printer.^112^ Herein, nanoparticles loaded with a Hippo pathway inhibitor, XMU-MP-1 within hydrogel demonstrated sustained release to promote nerve regeneration and functional restoration.

Moreover, conducting hydrogels encapsulating nanostructures have been employed for cardiac tissue engineering.^100^ As hydrogels are electrically insulating, incorporation of nanostructures such as carbon nanotubes (CNT) within gelatine methacryloyl (GelMA) hydrogels led to conducting nanohydrogel hybrid system along with strong mechanical properties without compromising its bioactivity, high porosity, and degradability.^113^ In another study, cardiac patches with improved mechanical stability and electrical coupling between cells were developed using CNT-incorporated GelMA hydrogels.^114^ These hybrid hydrogels with enhanced electrophysiological functions reported 3-fold higher spontaneous synchronous beating rates and an 85% lower excitation threshold with protective effects against a model cardiac inhibitor. Furthermore, an injectable CNT-functionalized reverse thermal gel (RTG) system with thermoresponsive properties was developed to replicate the unique electrophysiological property of native cardiac tissue.^115^ In addition, RTG systems display minimal swelling issues owing to their hydrophobic interactions-based fabrication. In a recent study, gold nanoparticles were incorporated in RTG system to provide topographical and electrophysiological cues for cardiac cell growth.^116^ In this nanoparticle–hydrogel culture platform, neonatal rat ventricular myocytes were co-cultured with cardiac fibroblasts and demonstrated good cell viability up to 21 days and high expression of predominant gap-junction protein, connexin 43 compared to an unmodified RTG hydrogel without nanoparticles.

### 3.3. Antibacterial resistance

**Fig. 3:**
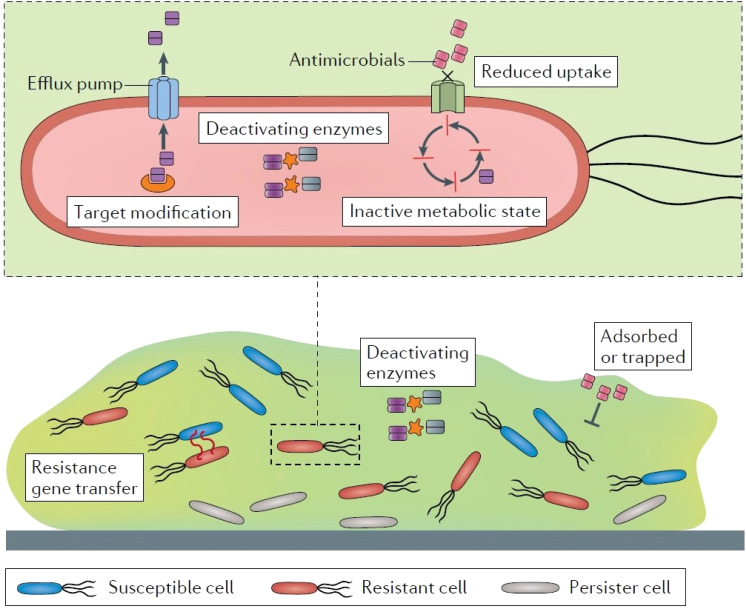
Antibiotic resistance mechanisms. Reproduced from ref. 136 with permission from Nature publishing group. This work is licensed under a Creative Commons Attribution 4.0 (CC BY) International License.

The infections caused by the bacteria can lead to antibacterial resistance due to overuse or improper use of antibiotics. The mechanisms through which bacteria acquire resistance include expulsion of drug *via* active efflux, prevention of drug entry, enzymatic inactivation of drug function, and mutation of targets (Fig. 3).^117–119^ Owing to these mechanisms, standard/conventional antimicrobial treatments are increasingly becoming ineffective for these resistant strains.^120,121^ Antibacterial resistance poses a severe threat to human health all over the world and demands urgent multisectoral action. In order to combat bacterial infections, antibacterial biomaterials such as hydrogels incorporating antimicrobial nanoparticles such as silver, gold, or copper have been developed as substitutes for antibiotics.^24,122^

Hybrid hydrogels-assisted delivery of non-antibiotic therapeutics (antibacterial nanoparticles, antibacterial agents, *etc.*) have greatly improved the treatment modalities for antibiotic-resistant bacterial infections and recurring infections. In addition, this hybrid system also enhances the effectiveness of existing antibiotics for therapeutics. Nanoparticle–hydrogel systems have gained much attention in recent years for their potential use in combating antimicrobial resistance due to their unique combination of properties. Nanoparticles can be used as carriers for drugs, while hydrogels can provide a sustained release of the drugs, thereby increasing their effectiveness. In addition, hydrogel provides a stable and biocompatible platform for the nanoparticles to release their antimicrobial agents.

The use of nanoparticle-incorporated hydrogels in the delivery of antimicrobial agents for the treatment of antibacterial resistance has several advantages, including:

• Improved drug bioavailability: nanoparticles are able to encapsulate the antimicrobial agents, which protects them from degradation, improves their stability, and enhances their bioavailability.

• Controlled release of the drug: the hydrogel matrix of the nanoparticle system can control the release of the antimicrobial agent, allowing for sustained release over an extended period of time.

• Targeted delivery: the nanoparticles can also be designed to specifically target the site of infection, thereby reducing the dosage required and minimizing the risk of systemic toxicity.

• Enhanced efficacy: the synergistic effect of combining the antimicrobial agents with nanoparticles can lead to increased efficacy against resistant strains of bacteria.

• Reduced toxicity: the use of nanoparticles can also reduce the toxicity associated with current antimicrobial agents by lowering the dosage required and minimizing exposure to healthy cells.

The mechanism of action of the nanoparticle–hydrogel system is thought to involve the physical interaction of the nanoparticles with the bacterial cell membrane, leading to membrane damage and ultimately cell death.^24,123^ Additionally, the hydrogel matrix in hybrid system may enhance the efficacy of the nanoparticles by increasing their contact time with bacteria. Here are some ways in which nanoparticle–hydrogel systems can combat antibacterial resistance:

• Directly targeting bacteria: nanoparticle-hydrogel systems can be designed to directly target bacteria. For instance, silver nanoparticles can be incorporated into hydrogel to create a wound dressing that releases antimicrobial silver ions. These systems have been shown to be effective against antibiotic-resistant bacteria such as Methicillin-resistant *Staphylococcus aureus* (MRSA).^124^

• Enhancing the effectiveness of antibiotics: nanoparticle-hydrogel systems can also be used to enhance the effectiveness of antibiotics. For example, hydrogels can be loaded with antibiotics such as vancomycin, and nanoparticles can be used to deliver the antibiotics directly to the site of infection.^125^ This can increase the local concentration of the antibiotic, making it more effective against bacteria.

• Preventing the formation of biofilms: antibiotic-resistant bacteria often form biofilms, which are protective communities of bacteria that are difficult to treat with antibiotics. Nanoparticle–hydrogel systems can be used to prevent the formation of biofilms. For instance, gold nanoparticles can be incorporated into a hydrogel to disrupt the attachment of bacteria to a surface, preventing the formation of biofilms.^126^

Hydrogels incorporating antibacterial nanoparticle's such as silver nanoparticles, gold nanoparticles, copper nanoparticles, zinc nanoparticles, and others have shown tremendous progress towards reducing the antibacterial resistance.^10,123^ These antibacterial nanoparticles exhibit broad antibacterial activity against *P. aeruginosa*, *Staphylococcus epidermidis*, *S. aureus*, *E. coli*, *Enterobacter cloacae*, *Proteus vulgaris*, *Proteus mirabilis, etc.*^14,127,128^

Among all these antibacterial nanoparticles, silver nanoparticles have been extensively utilized without causing any bacterial resistance. Hydrogels incorporating silver and gold nanoparticles inhibit the growth of bacteria either by destroying the pathogenic microorganisms or by preventing biofilm formation.^126,129^ Nanoparticles bind with negatively charged bacterial surface and biofilms electrostatically, influencing the exopolysaccharide production and bacterial growth.^130,131^ Various other studies suggest that the main mechanism of biofilm destruction using antibacterial nanoparticles occurs through the binding of silver or gold nanoparticles to the exopolysaccharide matrix. The biofilm structure destruction further leads to cellular membrane damage, DNA damage owing to the production of reactive oxygen species.^130,132^

The mechanism of action of other metal nanoparticles (Cu, MgO, FeO, ZnO, TiO_2_) incorporated hydrogels include cell membrane and DNA damage, production of active oxygen species, hydrogen peroxide formation *etc.*^133–135^ Overall, the use of nanoparticle-incorporated hydrogels in the delivery of antimicrobial agents has the potential to revolutionize the treatment of antibacterial resistance by improving drug bioavailability, reducing toxicity, and enhancing efficacy.

## 4. Experimental NPs incorporated hydrogels in delivery of the antimicrobial agents

NPs incorporated hydrogels for antimicrobial agents' delivery can be divided into various categories based on the types of encapsulated nanoparticles.^45,137,138^ The categories include hydrogel incorporated with antibacterial metallic nanoparticles, hydrogel incorporated with antibacterial polymeric nanoparticles, hydrogel incorporated with antimicrobial agents loaded nanoparticles, *etc.* Among antimicrobial agents/drugs that can be loaded into a hydrogel system include antimicrobial nanoparticles, antibiotics, biological extracts, antimicrobial peptides, *etc.* The properties of nanocomposite hydrogels in terms of promoting cell proliferation, and mechanical strength rely on the type of incorporated nanoparticles. Moreover, hydrogels incorporated with antimicrobial agents loaded nanoparticles display a tremendous increase in their antimicrobial efficacy. A comprehensive list of different antimicrobial agents loaded nanoparticle-hydrogel systems based on recent reports has been provided in Table 2. In this section, we have discussed different kinds of nanoparticle hydrogel composites along with their properties for controlling bacterial infection.

**Table 2:** Representative list of hydrogels loaded with different antimicrobial agents for controlling bacterial infections^a^

Nanocomposite material (hydrogel matrix/nanoparticles)	Type of antimicrobial agents	*In vitro*/*in vivo* studies	Bacteria	Findings	Ref.
Chitosan	Ag nanoparticles	*In vitro* and *in vivo* (rats)	*E. coli*, *S. aureus*	Excellent antibacterial performance (*E. coli*-99.86% and *S. aureus*-99.94%), enhanced re-epithelialization and collagen deposition, accelerated wound healing	127
Carboxymethyl chitosan and konjac glucomannan	Ag nanoparticles	*In vitro* and *in vivo* (rats)	*E. coli*, *S. aureus*	High antimicrobial activity, reduced inflammatory response	139
Chitosan quaternary ammonium salt, dextran, sodium hyaluronic	Ag nanoparticles	*In vitro* and *in vivo* (rats)	*E. coli*, *S. aureus*, *P. aeruginosa*	Excellent antibacterial ability with inhibition zone *E. coli*-24 mm, *S. aureus*-24 mm, and *P. aeruginosa*-27 mm with faster wound healing	140
Graphene oxide, pluronic F127, branched polyethyleneimine	Ag nanoparticles	*In vitro*	*E. coli*, *C. albicans*	Excellent antibacterial ability (*E. coli* 99.96% and *C. albicans*-99.94%)	141
Pluronic F127	Ag nanoparticles	*In vitro* and *in vivo* (rats)	*E. coli*, *S. aureus*, *P. aeruginosa*	Strong antibacterial activity and improved wound healing	142
Peptide (Phe_3_)	Ag nanoparticles	*In vitro*	*S. aureus*, MRSA	Enhanced antibacterial activity (3.12 mg L^−1^), significantly lowered MIC	143
PDEM	Ag nanoparticles	*In vitro* and *in vivo* (rats)	*E. coli*, *S. aureus*, *E. faecalis*	Good antibacterial ability, reduced toxicity, antioxidant properties, enhanced angiogenesis, and wound healing	144
Chitosan, gelatin	Au nanoparticles	*In vitro* and *in vivo* (rabbit)	MRSA	Efficient antibacterial effects, enhanced wound healing	145
Sodium alginate	Au nanoparticles	*In vitro* and *in vivo* (rats)	*E. coli*, *S. aureus*, *P. aeruginosa*	Bacterial killing-*E. coli*->95%, *P. aeruginosa*->95% and *S. aureus*-60%)	146
				Plate count method: *E. coli*->80%, *P. aeruginosa*->80% and *S. aureus*-35.4%)	
Silk fibroin, nHA	Ag and Au nanoparticles	*In vitro*	*S. aureus*, MRSA, *E. coli*, *S. epidermidis*	Significant inhibition ability against both Gram-positive and Gram-negative bacteria	147
Peptide (Nap-Phe-Phe-Tyr)	Au nanoparticles	*In vitro* and *in vivo* (rats)	*E. coli*, *S. aureus*	Significant inhibitory effect on both Gram negative and positive bacteria, rapid wound healing	148
Alginate, PVA	Au nanoparticles and pigallocatechin gallate	*In vitro* and *in vivo* (rats)	*E. coli*, *S. aureus*	Inhibited biofilm formation (*S. aureus*-94% and *E. coli*-92%), inhibition of plaque biofilm formation, enhanced bone regeneration	149
Carboxymethyl chitosan, hydroxyethyl cellulose	ZnO nanoparticles	*In vitro*	*E. coli*	pH responsive nanocomposite with significant inhibitory activity	150
Chitosan, elastin, sodium alginate	ZnO nanoparticles	*In vitro*	*E. coli*, *S. aureus*	Good antibacterial properties with varying concentrations of ZnO NPs, enhanced stem cells proliferation	151
Hyaluronic acid	ZnO nanoparticles	*In vitro* and *in vivo* (mouse)	*E. coli*, *S. aureus*	Enhanced ROS generation, improved *in vitro* and *in vivo* antibacterial performance	152
PAA and PVA	MgO nanoparticles	*In vitro*	*E. coli*, *S. aureus*	Higher antibacterial activity toward *S. aureus* than *E. coli*	153
Sodium alginate and polyacrylamide	Ag and MgO nanoparticles	*In vitro*	*E. coli*, *S. aureus*	Excellent mechanical properties, enhanced antibacterial effects with nanoparticles concentration, stimulation of osteogenesis	154
Cellulose, PAA	MgO nanoparticles, doxorubicin	*In vitro*	*E. coli*, *S. aureus*	pH triggered releases behavior, improved bactericidal activities in Gram positive bacteria compared to Gram negative bacteria, improved antitumor efficacy	155
Carboxymethyl chitosan	CuO nanoparticles	*In vitro*	*E. coli*, *S. aureus*	Higher swelling and excellent antibacterial behavior	156
PVA	Ag and TiO_2_ nanoparticles	*In vitro* and *in vivo* (rats)	*E. coli*, *S. aureus*	Light induced ROS generation, excellent antibacterial properties, enhanced biocompatibility, efficient prevention of *S. aureus* wound infection *in vivo*	157
PVA, starch, graphitic carbon nitride	Ag and TiO_2_ nanoparticles	*In vitro* and *in vivo* (rats)	*E. coli*, *S. aureus*	Capable of absorbing large volume of wound exudates, maximum zone of inhibition (*S. aureus*-33.25 mm and *E. coli*-37.33 mm), complete healing after 7 days	158
κ-carrageenan, chitosan, nHA nanoparticles	Antibiotics-ciprofloxacin	*In vitro*	*E. coli*, *S. aureus*	Sustained release of antibiotic (52–66% with high and low HA content), good antibacterial activities against both Gram positive and negative bacteria	159
Quaternized chitosan, hyperbranched nanoparticles	Antibiotics-clindamycin	*In vitro*	*E. coli*, *S. aureus*, MRSA	Dual pH responsive nanocomposites, excellent antibacterial properties (>90% bacterial killing)	160
Silk fibroin, PDA nanoparticles	Antibiotics-ciprofloxacin	*In vitro*	*P. aeruginosa*	pH responsive drug release, retention of >80% antibiotic bioactivity, inhibition of bacterial growth	161
Chitosan, sodium alginate, PVA	Ag NPs, SiO_2_ NPs, *Calendula officinalis* flower extract	*In vitro*	*E. coli*, *S. aureus*	Improved wound healing properties	162
Sodium carboxymethylcellulose, polyacrylamide, PDA nanoparticles	Biological extracts-vitamin C and curcumin	*In vitro* and *in vivo* (rat)	MRSA	Strong antibacterial activity against MRSA, improved wound closure, enhanced re-epithelialization, angiogenesis, and collagen deposition	163
Gum acacia, starch nanoparticles	Biological extracts-kaempferol	*In vitro*	*E. coli*, *S. aureus*	Retention of bioactivity for longer duration, excellent antimicrobial, antioxidant, antidiabetic properties	164
PEG, gold nanorods	Antimicrobial peptides-IRIKIRIK-CONH2 (IK8)	*In vitro*	*P. aeruginosa*, *S. aureus*	Photothermal triggered AMPs release, increased bactericidal activity with AMPs release and increased laser intensity	165
Zinc alginate, hollow silica nanoparticles	Antimicrobial peptides-RL-QN15	*In vitro* and *in vivo* (mice)	MRSA	Excellent biocompatibility, broad spectrum antimicrobial activity, accelerated re-epithelialization, granulation tissue formation	166

a Abbreviations: PAA-poly (acrylic acid), PVA-poly (vinyl alcohol), nHA-nano hydroxyapatite, PDEM-porcine dermal extracellular matrix, MRSA-methicillin resistant *S. aureus*, NPs-nanoparticles, MIC-minimal inhibitory concentration, ROS-reactive oxygen species, PDA-polydopamine, PEG-poly (ethylene glycol), AMPs-antimicrobial peptides, TCPP-tetra kis(4-ccarboxyphenyl) porphyrin.

### 4.1. Hydrogels incorporated with metal nanoparticles

**Fig. 4:**
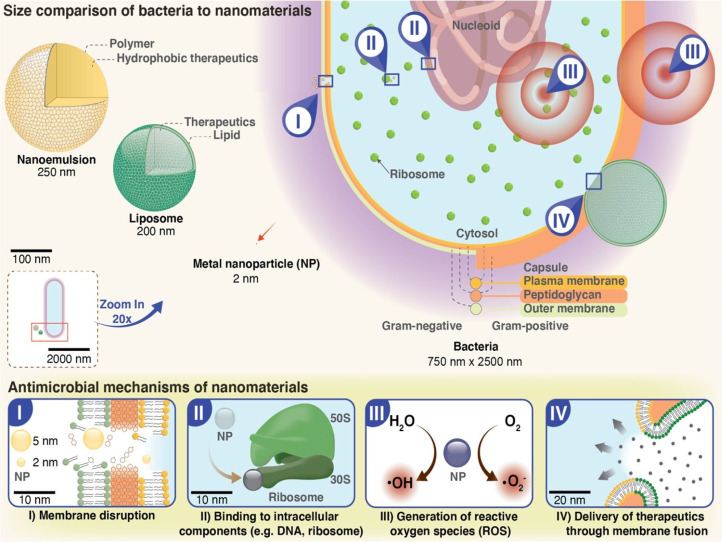
Size comparison between nanomaterials and bacteria (nano *versus* micro). Nanoparticles employ a variety of bactericidal mechanisms for killing or preventing bacterial infections. The mechanisms include (I) bacterial cell membrane disruption due to electrostatic interactions between negatively charged bacterial surfaces and NPs leading to cytoplasmic leakage. (II) Intracellular damage and interruption of their function due to binding of NPs with bacterial cells intracellular components such as DNA, ribosomes, and/or proteins. (III) Generation of reactive oxygen species (ROS) and oxidative cellular stress owing to the catalytic activities of NPs for increased ROS (hydroxyl radicals and superoxide's) production. (IV) Delivery of therapeutics through membrane fusion after rapid entry of NPs in bacterial cells. Reproduced from ref. 136 with permission from Nature Publishing group. This work is licensed under a Creative Commons Attribution 4.0 (CC BY) International License.

In order to control bacterial infections and the rise of resistance to commonly used antibiotics and disinfectants, development of new antimicrobial materials is pertinent to prevent infections caused by these pathogens. Hydrogels incorporated with metal nanoparticles for antimicrobial drug delivery is a promising approach for developing advanced materials that can effectively address the challenges associated with bacterial infections. There are various advantages of incorporating metal nanoparticles into hydrogels. The most important advantage of metal nanoparticles incorporation into hydrogels include their ability to present as a good substitute for antibiotics and rarely induce bacterial resistance despite their widespread use.^10^ The reason might be attributed to the multiple mechanisms of antimicrobial action by metal nanoparticles in contrast to only one mechanism of action utilized by antibiotics (Fig. 4). Other advantages include its small size, stable nature, and improved delivery system platform in order to bring sustainable antimicrobial effect to improve patient compliance while reducing the frequency of administration. Metal nanoparticles such as silver (Ag), gold (Au), copper (Cu), zinc oxide (ZnO), and titanium dioxide (TiO_2_) have been shown to exhibit broad-spectrum antimicrobial activity against various pathogens, including bacteria, viruses, and fungi.^24,167,168^ Hydrogels, on the other hand, have unique properties such as high water content, biocompatibility, and biodegradability, which make them suitable for biomedical applications. Hydrogels encapsulating metal nanoparticles can be prepared using various methods, including physical mixing, *in situ* synthesis, and surface modification. The choice of method depends on the type of nanoparticles and the hydrogel matrix utilized. Currently, various metallic nanoparticles (Ag, Au) and metal oxides nanoparticles (MgO, ZnO, and TiO_2_) with antimicrobial activity have been employed as a new choice of antimicrobials.^24,169^

#### 4.1.1. Hydrogels incorporated with silver nanoparticles

Among all employed metal nanoparticles, silver nanoparticles incorporated hydrogels are hot spots for researchers owing to their excellent antibacterial properties and reduced toxicity for overcoming antibacterial resistance. The utilization of silver as an antimicrobial dates back centuries for treatment of illness and extending shelf life of food substances owing to its bactericidal properties against microorganisms.^170^ In recent decades, silver in the forms of mainly silver nanoparticles (AgNPs) and AgNPs incorporated hydrogels have emerged as an efficient antibacterial agent towards wound treatment with the development of nanoscience and nanotechnology.^171^

Silver ion and silver nanoparticles have broad-spectrum antimicrobial activity against different microorganisms including resistant strains through action of Ag cation.^169,171^ Despite AgNPs has been utilized as an efficient antibacterial agent, their bactericidal mechanisms still remain unclear. Though, various hypothesis have been reported but the most accepted hypothesis states that the interaction of silver ion (Ag^+^) with the thiol group in proteins on the cell membrane helps in binding with the bacterial cell membrane, leading to inhibition of DNA replication, interference with bacterial cell's viability, and subsequent cell lysis.^172^ The antibacterial effects of AgNPs at present include disruption of cell membrane, inhibition of bacterial respiratory chain, induction of bacteria to produce oxidative stress to produce ROS, interference with bacterial protein synthesis and folding, induction of bacterial genotoxicity, and induction photocatalytic damage to bacterial proteins.^173^ In recent decades, various AgNPs have been synthesized which allows specific surfaced properties based on the type of reducing agents or stabilizers used during their synthesis.^169,174,175^ With the recent development in material engineering, AgNPs are loaded into degradable polymers, particularly hydrogels because the degradation process matches with the controlled release of AgNPs to achieve antimicrobial effect.^176^

AgNPs incorporated into hydrogels demonstrated excellent antibacterial properties and the ability to control bacterial infections. The morphology and size of the nanostructures are controlled by the polymer-based hydrogels by changing the amount of crosslinkers and monomers in the hydrogel network along with acting as a stabilizing medium.^177^ The loading of the NPs into porous hydrogels is carried out either by *in situ* polymer synthesis or by adding the NP colloid to the polymer. In addition, a microwave radiation-based approach is also being utilized for the incorporation of NPs within hydrogels. Hydrogels incorporating silver nanoparticles can be categorized into two types *viz.*, natural, and synthetic matrices based on the polymers utilized. The natural and synthetic polymers being utilized for fabrication of AgNPs loaded hydrogels include chitosan, carboxymethyl chitosan, sodium alginate, polyvinyl alcohol, hyaluronic acid, konjac glucomannan, carbopol-934, carboxymethyl cellulose, gelatin, polyvinylpyrrolidone, methacrylate, graphene, polyacrylamide, polyethyleneimine *etc.*^178–180^

Among all the explored polymers, polysaccharide chitosan has been extensively utilized for the fabrication of AgNPs loaded hydrogels that may be suggested due to the intrinsic antimicrobial activity of chitosan. The combination of AgNPs with polycationic chitosan has shown promising antimicrobial results by facilitating AgNPs attachment to the negatively charged bacterial wall.^181^ El-Sherif developed AgNPs-loaded hydrogel composed of poly (acrylamide-*co*-styrene sulfonic acid sodium salt) and chitosan to combat the most sensitive strains of *Bacillus subtilis* (*B. subtilis*) owing to good dispersion capability of AgNPs within the hydrogel network.^177^ Furthermore, multifunctional hydrogel system composed of AgNPs loaded chitosan-polyethylene glycol (CS-PEG) hydrogel system was explored for inhibiting biofilm formation with the release of AgNPs.^182^ Similarly, chitosan sponge containing AgNPs demonstrated excellent antibacterial activity against drug-resistant pathogenic bacteria and improved wound healing in both *in vitro* and *in vivo* studies.^183^ In another study, chitosan/graphene oxide-Ag nanocomposite hydrogel showed better antibacterial efficacy against Gram-negative bacteria and Gram-positive bacteria compared with the original chitosan hydrogels.^184^ Xie *et al.*, prepared CS-Ag hydrogel which displayed higher antibacterial efficacy and stronger compressive strength (100 times) than the pure chitosan hydrogels.^185^

The utilization of AgNPs as delivery vehicles is often limited by problems associated with its dispersion. The incorporation of AgNPs into hydrogels greatly reduced this shortcoming. In addition to circumventing nanoparticle dispersion issues, various additives such as counterion of positively charged Ag^+^ within the hydrogel matrix have shown good antimicrobial activity by decreasing the minimal inhibitory concentration (MIC) against Gram-positive bacterial and fungal (*S. cerevisiae*) strains while reducing the AgNPs toxicity.^186^ In another report, Mirzajani *et al.*, demonstrated the ability of AgNPs to disrupt the bacterial cell wall through their interaction with the peptidoglycan layer (breaking of β-1–4 bonds of *N*-acetylglucosamine and *N*-acetylmuramic acid of the glycan chain) in the *S. aureus* cell membrane.^187^ In another report, production of free radicals *viz.*, reactive oxygen species (ROS) inside and outside the bacteria resulted in good antimicrobial properties.^188,189^ Furthermore, alginate-based semi-interpenetrating polymer network (IPN) hydrogels incorporating AgNPs demonstrated good antibacterial activity.^190^

Jiang *et al.*, employed hydrogel derived from polysaccharide glucomannan konjac in combination with chitosan and silver nanoparticles as a wound dressing which exhibited the controlled release of silver nanoparticles.^139^ The developed hydrogel-AgNPs composites reduced inflammation and toxicity as well as absorbed the wound fluid. Furthermore, Haidari *et al.*, developed pH responsive AgNPs loaded smart hydrogel for sustained release.^191^ Herein, the pH changes from acidic to basic triggered the release of the AgNPs contained in the hydrogel and displayed effective elimination of Gram-positive and Gram-negative bacteria. In a recent report, microwave irradiation technology synthesized AgNPs loaded in chitosan-grafted polyvinyl alcohol hydrogels were utilized for wound healing.^192^ The reducing agent to synthesize AgNPs was a natural reducing agent, green tea leaf extract. The AgNPs loaded hydrogels exhibited excellent antibacterial activity against *E. coli* and *S. aureus* and displayed enhanced wound healing in both the *in vitro* (fibroblast cells) and the *in vivo* using rat models. Furthermore, a smart thermosensitive hydrogel and ROS-scavenging based on polydopamine modified poly(ε-caprolactone-*co*-glycolide)-β-poly(ethylene glycol)-β-poly(ε-caprolactone-*co*-glycolide) triblock copolymer loaded with AgNPs was developed for antimicrobial activities and wound healing.^193^ The *in vitro* and *in vivo* studies exhibited good antibacterial activity against *E. coli* and *S. aureus* and improved wound healing in *S. aureus*-infected rat model, respectively. The antibacterial response and wound healing of this AgNPs loaded composite hydrogel-based dressings was revealed through inhibition of bacterial growth, induction of angiogenesis and collagen deposition, and alleviation of the inflammatory response. Similarly, hydrogel-based wound dressings were fabricated using injectable sodium alginate (SA) hydrogel loaded with gallic acid (GA)-functionalized silver nanoparticles (AgNPs) for bacteria infected wound healing.^194^ The hydrogel dressings exhibited long-term antibacterial activity and biofilm formation reduction ability with sustainable release of silver ions. Subsequently, GA@AgNPs-SA hydrogel demonstrated accelerated wound healing of infected wounds through enhanced angiogenesis, and reduced inflammation, as revealed through decrease of IL-6 and TNF-α expression and upregulation of CD31, α-SMA and VEGF expression.

Although AgNPs-based hydrogels have extensively been utilized for antimicrobial properties, its stability (physical and chemical instability), safety, and efficacy (more effective to Gram − bacteria compared to Gram + bacteria are still far from what is expected).^195^ The less effectiveness against Gram + bacteria occurs due to high resistance of peptidoglycan layer of the bacterial cell wall. Therefore, several vital issues such as antimicrobial ability against Gram-positive bacteria, reduction of serum albumin, and minimization of gene toxicity should be considered when designing AgNP-based hydrogels.^196^ To attempt this, various environmentally friendly synthesis strategies have been employed to improve their properties to ensure the clinical potential of the AgNPs loaded hydrogels.^191,197^

#### 4.1.2. Hydrogels incorporated with gold nanoparticles

In addition to AgNPs, gold (Au) nanoparticles also exhibit antimicrobial properties, which otherwise are absent in bulk or ionic gold.^198,199^ In addition to antimicrobial properties, tunable core size, biocompatibility, photothermal and photodynamic properties, high chemical stability, localized surface plasmon resonance (LSPR) properties, high X-ray absorption coefficient, and efficiency in generating ROS make AuNPs suitable for wide range of applications.^24,200^ The antimicrobial mechanism of Au nanoparticles includes attachment to bacterial cell, and disruption of peptidoglycan layers leading to the leakage of bacterial contents. AuNPs when combined with non-antibiotic or antibiotic molecules also has capability to reverse bacterial resistance to some extent.^201^ Daniel-da-Silva *et al.* developed a gelatin-based stimuli (temperature) responsive hydrogel loaded with gold nanoparticles.^202^ The Au/gelatin hydrogel nanocomposites released Au nanoparticles under the influence of thermal stimuli and promoted bone regeneration without any infections or toxicity. In other study, Jayaramudu *et al.*, developed biodegradable gold nanocomposite hydrogels for antibacterial applications.^203^ The hydrogel was derived from acrylamide (AM) and wheat protein isolate (WPI) and demonstrated effective antimicrobial effects. Furthermore, hydrogel loaded with Au NP-stabilized liposomes demonstrated excellent antibacterial properties against *S. aureus* without skin toxicity.^204^ Ribeiro *et al.*, utilized silk fibroin/nanohydroxyapatite hydrogel incorporating AuNPs which displayed antimicrobial activity and promoted bone regeneration.^147^ In another study, potent *in vitro* antibacterial activity against *S. aureus* and *P. aeruginosa* was reported using hydrogels (prepared using polyethylene glycol (PEG) loaded with gold nanorods (AuNRs) and cationic poly allyl amine hydrochloride (PAH).^205^ The nanocomposite hydrogel demonstrated slow and prolonged release behaviour, and remarkable wound healing properties in *in vitro* and *in vivo* studies. In a recent report, hydrogel loaded with Au nanoparticles and epigallocatechin gallate (EGCG) displayed bactericidal activity and periodontal tissue regeneration.^149^ The synergistic antimicrobial and photothermal properties (NIR irradiation for 5 minutes) exhibited by multifunctional hydrogel effectively inhibited the *E. coli* and *S. aureus* biofilms by 92% and 94%, respectively. Thereafter, *in vivo* studies in rat periodontitis model demonstrated inhibition of dental plaque biofilm formation and promotion of alveolar bone regeneration by 87% and 38%, respectively. Moreover, gold nanoparticles-loaded hydrogel-based wound dressings have been developed in recent years for improvement of wound healing and controlling bacterial infections. Chen *et al.*, developed supramolecular hydrogels Nap-Phe-Phe-Tyr (NapFFY) loaded with gold nanoparticles for antibacterial and wound healing applications.^148^ The continuous and sustained release of Au nanoparticles from Au@hydrogel based wound dressing promoted rapid wound healing (shorter healing time) and controlled *E. coli* and *S. aureus* growth compared to AuNPs or hydrogels alone without causing any toxicity. The antimicrobial and rapid wound healing capability presents this Au-hydrogel as a suitable candidate for clinical trials in the future.

#### 4.1.3. Hydrogels incorporated with other metal nanoparticles

Apart from primarily reported silver and gold nanoparticles encapsulated hydrogels, several other metallic nanoparticles (NPs)-hydrogel composite also show promise for controlling bacterial infections and wound healing. Although, the mechanisms are not clearly known, the general antibacterial mechanisms employed by metallic nanoparticles include damage to peptidoglycan and/or cell membrane, damage to DNA/inhibition of DNA transcription, inhibition of electron transport chain, damage/inhibition of ribosomes, damage/inhibition of proteins *etc.*^206^ Kumar *et al.*, demonstrated excellent antibacterial activity using chitin hydrogel loaded with nickel nanoparticles against *S. aureus*.^207^ In another study, cobalt-exchanged natural zeolite/PVA hydrogel showed efficient antibacterial activity against Gram-negative bacteria, (*E. coli* and *S. aureus*).^208^ Furthermore, copper-based nanoparticles have been employed for antimicrobial properties. Although copper nanoparticles have weaker antibacterial effects than silver nanoparticles, they possess broad antimicrobial properties against both bacteria (*E. coli*, *S. aureus* and *Listeria monocytogenes*) and fungi (*Saccharomyces cerevisiae*).^209–211^ In other studies, carboxymethyl cellulose and chitosan hydrogels loaded with Cu nanoparticles demonstrated excellent antibacterial effects against *E. coli* and *S. aureus* without causing any toxicity.^212,213^ Villanueva *et al.*, effective antimicrobial activity was observed against Gram-negative and Gram-positive bacterial species using a starch hydrogel loaded with different concentrations of CuNPs.^214^ Despite utilization of these metallic nanoparticles, there is still need for further designing and exploration of other metallic nanoparticles-hydrogels composite for antimicrobial activities.

#### 4.1.4. Hydrogels incorporated with metal oxide nanoparticles

In addition to the hydrogels loaded with different metal nanoparticles, hydrogels encapsulated with metal oxide nanoparticles also possess good antibacterial properties. In contrast to metal nanoparticles, the main antibacterial mechanism of metallic oxide nanoparticles includes photocatalysis.^215^ Among the various available metallic oxides-based nanoparticles, zinc oxide (ZnO) represents the most popular due to its strong antimicrobial activity and low cytotoxicity.^216^ The antimicrobial mechanisms of ZnO NPs rely mainly on two aspects for combating microbes. (1) Formation of Zn^2+^ ions and reactive oxygen radicals including hydrogen peroxide (H_2_O_2_),^217,218^ and (2) tight binding of ZnO NPs to bacterial cell membranes for disruption of proteins and lipids, increased membrane permeability and cell lysis.^206,217,219^ In an earlier study, Mohandas *et al.*, developed a composite bandage of sodium alginate hydrogel loaded with ZnO NPs for controlling bacterial infections and wound closure. The hydrogel/ZnO NP composite bandage demonstrated excellent antimicrobial activities against *S. aureus*, *E. coli*, *Candida albicans*, and methicillin-resistant *S. aureus* without causing any toxicity.^220^ In other study, carboxymethylcellulose (CMC)/ZnO nanocomposite hydrogel synthesized *via in situ* polymerization exhibited antibacterial effects against *E. coli* and *S. aureus* bacteria.^221^ Apart from natural polymer-based hydrogel, PEG methyl ether methacrylate modified ZnO (ZnO-PEGMA) and 4-azidobenzoic agarose (AG-N3) nanocomposite hydrogels with inter-penetrating network structure demonstrated excellent bactericidal activity and anti-adhesive property against Gram-negative *E. coli* and Gram-positive *S. aureus*.^222^

Furthermore, antimicrobial hydrogels based on poly *N*-isopropylacrylamide (PNIPAM) loaded with ZnO NPs have shown bactericidal activity against *E. coli*.^223^ Apart from ZnO, magnesium oxide (MgO), titanium oxide (TiO_2_), and iron oxide (FeO) nanoparticles loaded with hydrogel have been utilized for efficient antibacterial activities. Archana *et al.* developed TiO_2_ nanoparticles-loaded chitosan–pectin composite hydrogel wound dressings which showed good antibacterial ability, photoactive properties and enhanced wound closure rate in the excision wound model.^224^ In another study, gellan gum biopolymer hydrogel film with Ag-loaded TiO_2_ nanorods (Ag@TiO_2_NRs/GG) based wound healing dressing was developed for evaluation of skin tissue regeneration.^225^ The results of this study reported 100% wound healing using Ag@TiO_2_NRs/GG hydrogel as revealed through clear dermis, thicker epidermis, and subcutis layer after 14 days of treatment. Furthermore, MgO nanoparticles doped cellulose hydrogel were developed for pH triggered sustained release of doxorubicin and demonstrated efficient antibacterial and anti-cancer effects.^155^ In another study, Saravanakumar *et al.*, developed xanthan gum-based nanocomposite loaded with aloe vera extracts and bimetallic (Ag and MgO) nanoparticles. The developed nanocomposites displayed higher antibacterial activity against *B. cereus* and *E. coli* with zone of inhibition of 15.00 ± 0.12 mm and 14.50 ± 0.85 mm, respectively. Furthermore, the *in vivo* studies demonstrated higher wound closure activities compared to control without nanoparticles.^226^ In a recent study, chitosan/polyvinyl alcohol hydrogels loaded with FeO nanoparticles for investigation of antimicrobial properties and wound healing. Herein, the nanocomposite hydrogels demonstrated efficient control over bacterial infections in treatment of diabetic foot infections.^227^

Overall, hydrogels encapsulating metal nanoparticles have shown promising results for controlling bacterial infections. In addition, hydrogels encapsulating metal nanoparticles can also serve as a platform for combining multiple antimicrobial agents, such as antibiotics, peptides, and biological extracts, to develop advanced materials that exhibit enhanced antimicrobial activity. However, it is important to optimize the properties of these materials, including their mechanical strength, stability, morphology, particle size, surface properties, associated signal transduction mechanisms, and biocompatibility, to ensure their safety and efficacy in different applications.^228^ In addition, further studies are needed to investigate the potential toxicity of metal nanoparticles and their effects on the environment as toxicities of the metal-based nanomaterials still remain a major concern despite being used in low concentrations. Nevertheless, hydrogels encapsulating metal nanoparticles for antimicrobial drug delivery are a promising approach that can potentially address the challenges associated with bacterial infections and improve patient outcomes.

### 4.2. Hydrogels incorporated with polymeric nanoparticles

Hydrogels encapsulating polymeric nanoparticles for antimicrobial drug delivery is a promising approach for developing advanced materials that can effectively address the challenges associated with bacterial infections. Hydrogels have unique properties such as high water content, biocompatibility, and biodegradability, which make them suitable for biomedical applications. On the other hand, polymeric nanoparticles, such as chitosan, poly (lactic-*co*-glycolic acid) (PLGA), and polyethylene glycol (PEG), have been shown to exhibit antimicrobial activity against various pathogens, including bacteria, viruses, and fungi.^17,229,230^ Among all the polymers utilized, chitosan nanoparticles with cationic characteristics confers powerful antimicrobial effects through interaction with the negatively charged microorganisms cell membranes.^231,232^ Hydrogels encapsulating polymeric nanoparticles can be prepared using various methods, including physical mixing, *in situ* synthesis, and surface modification. The choice of method depends on the type of nanoparticles and the hydrogel matrix used. The incorporation of polymeric nanoparticles into hydrogels can enhance their antimicrobial activity, improve their stability, and provide sustained release of the nanoparticles, which can reduce the frequency of administration and improve patient compliance. In addition, hydrogels encapsulating polymeric nanoparticles can also serve as a platform for combining multiple antimicrobial agents, such as antibiotics, peptides, and biological extracts, to develop advanced materials that exhibit enhanced antimicrobial activity.

**Fig. 5:**
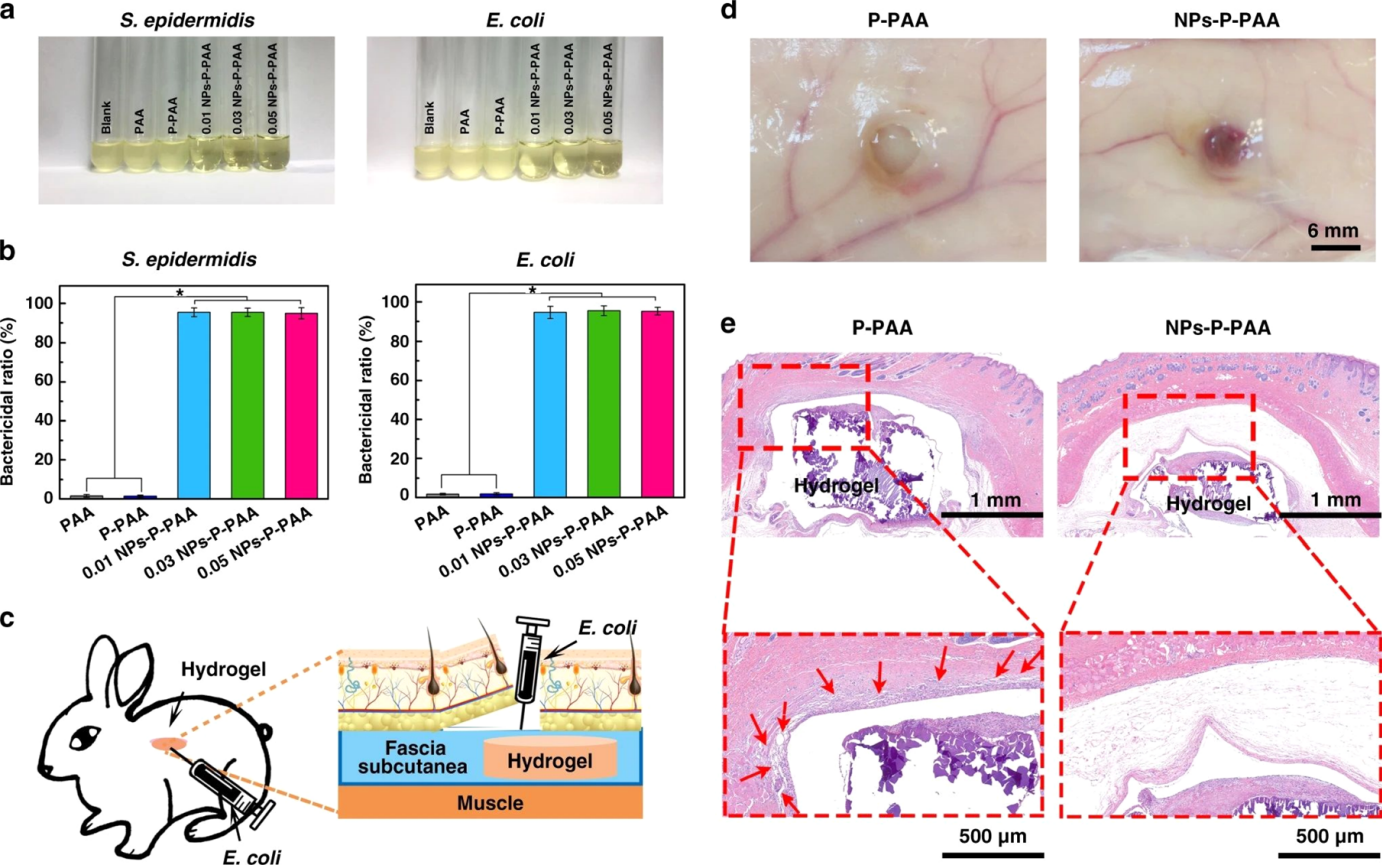
The antibacterial activity of plant inspired tough hydrogel. (a) Co-culture of *S. epidermidis* and *E. coli*. solution with hydrogels, (b) hydrogels bactericidal ratio against *S. epidermidis* and *E. coli*. (c) *In vivo* antibacterial experiments design. (d) Harvested hydrogels post-surgery. (e) Histological images showing connective tissues surrounding the hydrogel. Reproduced from ref. 233 with permission from Nature publishing group. This work is licensed under a Creative Commons Attribution 4.0 (CC BY) International License.

Zhang *et al.* developed a bioadhesive NPs-loaded hydrogel (NLH) for the local delivery of a broad-spectrum antibiotic ciprofloxacin to control bacterial infections.^78^ In this study, hydrogel was composed of dopamine methacrylamide, polyethylene glycol (PEG) dimethacrylate, and polyvinyl alcohol (PVA). The fabricated hydrogel encapsulated ciprofloxacin loaded PLGA NPs for comparison of release profile with or without nanoparticles. The NLH showed a controlled and sustained ciprofloxacin release profile in contrast to burst release profile displayed by the blank hydrogel without nanoparticles. The *in vitro* antibacterial efficacy of different formulations such as free drug, drug-NPs, blank hydrogel, and drug-loaded NLH demonstrated varied results. Unlike the other formulations, the ciprofloxacin-NLH completely inhibited the biofilm formation by *Escherichia coli*. Furthermore, the adhesive capabilities of the ciprofloxacin-NLH on different biological surfaces indicated a large quantity of NPs retention without causing any toxicity. In another study, a plant inspired tough and adhesive hydrogel (pectin and polyacrylic acid) loaded with Ag–Lignin NPs demonstrated strong bactericidal activity owing to their ability to generate free radicals for improved wound repair (Fig. 5).^233^ The incorporation of Ag–Lignin NPs into hydrogel improved the mechanical properties and adhesive properties of the hydrogel. Furthermore, Shafique *et al.*, developed polymeric nanoparticles loaded hydrogel (NLH) with antibacterial properties for wound healing.^234^ Herein, the hydrogel was composed of hyaluronic acid (HA), pullulan, and PVA which are incorporated with water-insoluble hydrophobic drug, cefepime, loaded chitosan nanoparticles. The developed NLH acts against Gram-positive (*Staphylococcus aureus*, *Pseudomonas aeruginosa*) and Gram-negative bacteria (*Escherichia coli*) which allows sustained release of cefepime along with good stability and swelling capacity. The drug-NLH showed accelerated wound healing process with a wound closure rate of 100% after 14 days compared to cefepime in solution with a wound closure rate of 80%.

The improved wound healing using developed NLH was displayed by the presence of hair follicles, absence of inflammatory cells, and presence of sweat, and sebaceous glands at the wound site. In order to ensure proper wound healing, keeping a wound from becoming infected presents a crucial feature. Zeng *et al.*, fabricated xanthan gum and konjac glucomannan-derived hydrogel loaded with polydopamine NPs to heal bacteria-infected wounds.^235^ The developed NLH exhibited broad-spectrum antibacterial activity against Gram-negative (*Escherichia coli*) and Gram-positive (*Staphylococcus aureus*) bacteria indicated through *in vitro* near-infrared photothermal antibacterial experiments. Furthermore, *in vivo* studies in rats demonstrated improved wound healing as displayed by improved collagen deposition, formation of new hair follicles, promotion of vascular angiogenesis, significantly lower expression of pro-inflammatory cytokines and greater regularity of both epithelium and connective tissue. In another study, Wang *et al.* developed a promising approach for treatment of bacterial infections present either as open or subcutaneous wounds.^236^ In this study, a polyacrylamide (PAM) hydrogel encapsulated with platensimycin loaded polyamidoamine (PAMAM) NPs was developed to treat *Staphylococcus aureus* infections without inducing any toxicity to murine macrophage cells. The platensimycin-loaded NPs incorporated in hydrogel exhibited better controlled release behaviour than the free drug and platensimycin-loaded NPs. In a recent study, on-demand delivery-based hybrid hydrogel system using lipase-sensitive fusidic acid (FA) polymeric poly (ε-caprolactone) nanoparticles-loaded hydrogel was explored against MRSA-infected burn wounds.^230^ The fabricated FA-NPs exhibited lipase-sensitive and sustained release behaviour with a 2–4-fold increase in their antibacterial activity against chronic wound infection. Furthermore, *in vivo* potential of these developed systems was assessed in an MRSA-induced wound infection model, which demonstrated rapid and fast reepithelization, huge decrease in bacterial count, and higher rate of wound contraction. These results report a unique on-demand and site-specific release of FA at the wound site for preventing wound bacterial infection. Moreover, FA-NPs hydrogel formulation presents an alternative to commercial cream for controlling wound infections, especially in MRSA infected wounds. Despite of promising antimicrobial properties of different polymeric nanoparticles loaded hydrogels, it is important to optimize the properties of these materials, including their mechanical strength, stability, and biocompatibility, to ensure their safety and efficacy in different applications. In addition, further studies are needed to investigate the potential toxicity of polymeric nanoparticles and their effects on the environment. Overall, hydrogels loaded with polymeric nanoparticles for antimicrobial drug delivery are a promising approach that can potentially address the challenges associated with bacterial infections and improve patient outcomes.

### 4.3. Hydrogels incorporated with antibiotics-loaded nanoparticles

Hydrogels incorporated with antibiotics have been earlier reported as a promising drug delivery system for bacterial infections. However, with the direct incorporation of antibiotics into hydrogels quick initial burst release of antibiotics occurs which hinders the formation of local persistent high-concentration antibiotic environment due to sub-inhibition concentration of antibiotics after initial rapid release.^159,160,237^ Moreover, sub-inhibition concentration of antibiotics in local environment due to long-term slow release of antibiotics after initial burst release demonstrated a strong restructuring effect on a bacterial genome's functionality, leading to resistance to antibiotics.^238,239^ In order to circumvent these issues, different drug carriers have been developed which allow sustained release of antibiotics.^240,241^ Among different drug carriers, nanoparticles-based drug carriers have garnered much attention in recent years for delivery of different antibiotics.^242^ In addition, nanoparticles-based drug carriers can exhibit stimuli responsive release behaviour according to different environments and also allow delivery of hydrophobic antibiotics which was otherwise difficult by using hydrogels only.^25^ Furthermore, these advantages revealed the possibility of combining nanoparticles with hydrogel for overcoming quick initial burst release and antibiotic resistance problems encountered with hydrogels alone. Hydrogels incorporating antibiotics-loaded nanoparticles are a promising approach to enhance the antimicrobial activity of hydrogels and improve their efficacy for various biomedical applications, such as wound healing, tissue engineering, and drug delivery.^243^ Nanoparticles loaded with antibiotics can be embedded within the hydrogel network using different methods, including physical entrapment, covalent bonding, or electrostatic interaction. The choice of method depends on the type of nanoparticles and the hydrogel matrix utilized. Moreover, the incorporation of antibiotics-loaded nanoparticles into hydrogels can improve their antibacterial activity, increase their stability, and provide sustained release of the antibiotics, which can reduce the frequency of administration and improve patient compliance.

In an earlier study, Alvarez *et al.*, developed silica nanoparticles/collagen nanocomposites to evaluate gentamicin release as novel drug delivery systems to prevent infection in chronic wounds.^244^ Herein, collagen hydrogel incorporating gentamicin-loaded silica nanoparticles exhibited prolonged antibacterial activity against *Pseudomonas aeruginosa* and *Staphylococcus aureus*. In another study, an adhesive PLGA nanoparticle–hydrogel hybrid system based on marine mussels was developed for localized antimicrobial drug delivery of ciprofloxacin to inhibit the formation of *Escherichia coli* films.^78^ The nanoparticle–hydrogel composite provided effective antibiotic delivery and remained intact on a bacterial biofilm, mouse skin tissue and a mammalian cell monolayer. Furthermore, chitosan/κ-carrageenan based hydrogels incorporating ciprofloxacin-loaded hydroxyapatite (HA) nanoparticles have been developed for sustained release ciprofloxacin against Gram-positive *S. aureus* and Gram-negative *E. coli* bacteria. The hydrogel nanocomposites released only 52 and 66% of the loaded antibiotic with high and low content of HA, respectively.^159^ In contrast, native ciprofloxacin hydrogels (without NPs) released about 98% of ciprofloxacin during 120 h. In another study, dual crosslinked nanocomposite hydrogels based on quaternized chitosan and clindamycin-loaded hyperbranched nanoparticles demonstrated an excellent antibacterial activity (∼90%) not only against *S. aureus* and *E. coli*, but also against Methicillin-resistant *S. aureus* (MRSA).^160^ In recent years, various research groups have developed and employed hydrogels encapsulated with antibiotics-loaded nanoparticles for different bacterial infection treatment.

Karimi *et al.*, developed wound dressings based on carboxymethyl chitosan/sodium carboxymethylcellulose/agarose hydrogel incorporating silk fibroin/polydopamine nanoparticles for antibiotic delivery.^161^ In this study, three different ciprofloxacin-loaded nanoparticles *viz.*, silk fibroin nanoparticles (SFNPs) loaded with ciprofloxacin, SFNPs blended with Polydopamine (SFPDA NPs) loaded with ciprofloxacin, and SFNPs modified with Polydopamine (SFNPs@PDA) loaded with ciprofloxacin were synthesized and incorporated into the hydrogel. The sustained release of ciprofloxacin was effective in inhibiting the growth of *Pseudomonas aeruginosa* while preserving more than 80% bioactivity. The antibiotic release was more at alkaline pH (up to 75%) compared to acidic (∼50%) and neutral (∼55%) conditions. In another study, hydrogels incorporating antibiotic loaded solid lipid nanoparticles (SLNs) was utilized for co-delivery of adapalene and minocycline for acne treatment.^245^*In vitro* release study showed prolonged release of both drugs and exhibited antibacterial property comparable to market formulation containing clindamycin and adapalene. Furthermore, Rani *et al.*, developed hydrogel nanocomposites based on carboxymethyl tamarind kernel gum (CMTKG)/poly (sodium acrylate) and zinc oxide (ZnO) nanoparticles for oral delivery of ciprofloxacin drug and their antibacterial properties.^246^ The antimicrobial action of ciprofloxacin-loaded CMTKG-based hydrogels was enhanced with the incorporation of ZnO NPs. The release kinetic modelling of drug release was performed using Korsmeyer–Peppas model followed by Fickian diffusion. The kinetic modelling of antibiotic release using different models revealed the mechanistic pathway of drug release from the hydrogels with higher value of regression coefficient (*R*^2^) close to unity. Despite the use of antibiotics loaded nanoparticles within hydrogels for combating bacterial infections, still remain some limitations. The potential drawbacks of NPs–hydrogel system with antibiotics include possibility of development of antibiotic resistance. Therefore, it is crucial to optimize the properties of utilized materials, including their release kinetics and biocompatibility, to ensure their safety and efficacy in different applications. Overall, hydrogels incorporating antibiotics loaded nanoparticles offers a promising approach that can potentially address the challenges associated with bacterial infections and improve patient outcomes.

### 4.4. Hydrogels incorporating with biological extracts-loaded nanoparticles

Biological extracts loaded nanoparticles can be obtained from natural sources such as plants, animals, or microorganisms. The most studied biological extracts derived bioactive chemical compounds include polyphenols, flavonoids, alkaloids, tannins, and terpenoids that have a wide range of biological activities, including antioxidant, anti-inflammatory, and antimicrobial properties.^247^ Biological extracts are mostly of natural origin; therefore, they are considered safe and utilized as an alternative to chemical antibiotics for antimicrobial activities. Biological extracts often exhibit excellent biocidal properties and thus can be utilized for antimicrobial drug delivery. However, poor bioavailability and lack of targeting capacity limit its applications. To overcome these problems, biological extracts are encapsulated in nanoparticles and then incorporated within hydrogels. Hydrogels containing biological extracts-loaded nanoparticles enhance biological properties of extracts, such as antibacterial, antifungal, antiviral, antioxidant, and anti-inflammatory activities.^248^ Hydrogels incorporated biological extracts loaded nanoparticles can be prepared using various methods, such as physical entrapment, covalent bonding, or electrostatic interaction. The choice of method depends on the type of nanoparticles and the hydrogel matrix used. The incorporation of biological extracts loaded nanoparticles into hydrogels can enhance their biological activity, making them useful for wound healing, tissue engineering, and drug delivery applications. These materials have the potential to improve patient outcomes and reduce healthcare costs associated with various diseases. However, further research is needed to optimize the properties of these materials, including their mechanical strength, stability, and biocompatibility, to ensure their safety and efficacy in different applications. In addition, it is important to ensure that the extraction methods used are sustainable and environmentally friendly.

Quercetin is typical flavonoids which possess strong antioxidant properties and are extensively explored for wound healing applications. Jangde *et al.*, fabricated and evaluated quercetin loaded multiphase hydrogel comprising of carbopol liposomes as functional wound dressings for wound.^249^ The *in vitro* and *in vivo* evaluation results demonstrated accelerated wound-healing with sustained release of quercetin, as revealed through significant decrease in wound closure time as compared to conventional drug delivery systems. In another study, chitosan-cellulose hydrogel incorporating quercetin loaded with green zinc oxide nanoparticles were developed aimed at controlled release and improving bioavailability of quercetin for antimicrobial activities.^250^ In this study, quercetin and zinc oxide nanoparticles (ZnO NPs) were phyto-derived from onion peel waste and musk melon seeds, respectively. The nanohybrid hydrogels reported inhibition of the growth of *Staphylococcus aureus* and *Trichophyton rubrum* strains similar to commercial quercetin with the controlled and sustained release of phyto-derived quercetin.

Essential oils are biological extracts derived from plants that are widely known for their many health benefits including aromatherapy, and antimicrobial properties.^251^ Various essential oils naturally possess antimicrobial compounds such as aldehydes and phenols to fight off pathogens. Buntum *et al.*, evaluated the potential of semisolid PVA hydrogels incorporating essential oil loaded chitosan nanoparticles for wound management.^252^ In this study, two types of essential oils *viz.*, clove essential oil (CEO) and turmeric essential oil (TEO) were incorporated within nanoparticle–hydrogel system to prolong the release rate. The slow and sustained release of essential oils from hydrogel nanocomposite system presents its suitability for long-term antibacterial effectiveness.

Biological extracts loaded nanoparticles within hydrogel system have been utilized for chronic wound healing applications. Rodríguez-Acosta *et al.*, developed chitosan-based hydrogels incorporating calendula extract loaded silver nanoparticles and evaluated their antibacterial, healing, anti-infective, hemostatic, and anti-inflammatory functions through controlled release of calendula extract and nanoparticles.^253^ The combined delivery of silver nanoparticles and calendula extract demonstrated concentration-dependent antibacterial behaviour against *S. aureus* and *E. coli*, indicating suitability for chronic wound healing. In another study, hydrogel wound dressings were developed utilizing *Calendula officinalis* flower extract loaded antibacterial nanoparticles (silicon dioxide and silver nanoparticles) within three-component chitosan (CS)/sodium alginate (SA)/polyvinyl alcohol (PVA) hydrogel for wound healing application.^162^ Furthermore, in another study, tea polyphenol epigallocatechin gallate (EGCG) was loaded into gold nanoparticle-modified hydrogels for inhibition of oral bacterial infection and periodontal disease bone repair.^149^ Herein, the multifunctional hydrogel nanoplatform achieved the synergistic functions of near-infrared (NIR) photosensitization and bactericidal properties by inhibiting bacteria and inducing bone regeneration by promoting angiogenesis. The NIR-irradiated composites inhibited *E. coli* and *S. aureus* biofilms formation in *in vitro* studies by 92%, 94% with 5-fold increase in alkaline phosphatase activity and 3-fold increase in extracellular matrix mineralization rate. The *in vivo* studies in rat periodontitis model demonstrated induced alveolar bone regeneration (38%) and inhibition of dental plaque biofilm (87%). In a recent study, resistant starch nanoparticle-gum acacia hydrogel was utilized for controlled release of flavonoids, kaempferol.^164^ The toxicological and nutraceutical evaluation of hybrid hydrogels demonstrated enhanced bioavailability and bioactivity retention of kaempferol in human digestive conditions.

Curcumin has been utilized for centuries as a remedy for various ailments owing to its nontoxic nature and bioactivity. However, its widespread applications in drug delivery systems are limited by its poor bioavailability and solubility in aqueous solutions. Therefore, integrating curcumin with nanoparticle-loaded hydrogels system would provide sustained and controlled release for long period of time along with improved bioavailability. In a recent study, Babaluei *et al.*, developed an injectable hydrogel–nanocomposite composed of sodium carboxymethylcellulose/polyacrylamide/polydopamine containing vitamin C and curcumin as wound dressings with antioxidant and antibacterial properties.^163^ The nano component of multiphase hydrogel system includes silk fibroin/alginate nanoparticles to reduce bacterial infection and enhance wound regeneration. The multiphase functional hydrogel demonstrated antibacterial activity against methicillin-resistant *Staphylococcus aureus* (MRSA) and supported superior full-thickness burn regeneration. The hybrid hydrogels showed improved re-epithelialization, wound closure, and collagen expression in the preclinical study along with neovascularization and anti-inflammatory effects. Overall, the hydrogels incorporated with biological extracts-loaded nanoparticles are a promising material for local and sustained delivery of therapeutics that can promote wound healing and tissue regeneration.

### 4.5. Hydrogels incorporated with antimicrobial peptides-loaded nanoparticles

Antimicrobial peptides (AMPs) are naturally occurring class of short polypeptides (∼12–50 amino acid residues) that exhibit a broad spectrum of antimicrobial activity against bacteria, viruses, and fungi.^254^ AMPs have gained wide interest in recent years as potential alternatives to conventional antibiotics due to their excellent antimicrobial properties and limited tendency towards antibiotic resistance development. Moreover, AMPs are considered an attractive future alternative to traditional antibiotics owing to their ability to penetrate the bacterial cell membrane and enter into intracellular targets for antibacterial/antibiofilm applications.^255,256^ However, its clinical translation has been limited by its susceptibility to protease degradation resulting in a shorter effective time and unstable antibacterial effects.^257^ Therefore, it is important to develop a suitable delivery system to improve the bioavailability, stability, and targetability along with its sustained release for effective antimicrobial effects. To attempt this, nanocarriers loaded AMPs delivery have been explored to enhance antimicrobial effectiveness and minimize the chances of resistance development. Nanoparticles-based AMP delivery serves as emerging option for effective treatment of bacterial infections as NPs can protect AMPs from protease, improve their stability, and deliver them to targeted area. For instance, Primo *et al.* recently developed the AMP Ctx(Ile^21^)-Ha-conjugates with rifampicin (RIF)-loaded NPs in order to improve the physiochemical stability, antimicrobial effect, and resistance.^258^ The findings of the study suggested that AMP-conjugated RIF-loaded CS-based NPs were efficient for the delivery RIF (anti-tuberculum drug) into macrophages with 100% bioavailability. Moreover, these NPs exhibited excellent antibacterial activity in MDR clinical isolated strains of the MTB suggesting the potential of the AMP-nanocarriers systems to overcome MDR in bacteria. Similarly, many other AMP nanoparticle systems have been investigated to protect AMP, and improve their bioavailability and safe delivery into targeted sites. These AMP-NPs systems have been comprehensively reviewed recently.^259^ However; still remains some shortcomings associated with the NPs *viz.*, toxicity, nanocarrier aggregations, and non-biodegradability. In order to circumvent these issues, improved drug delivery systems need to be developed. Hybrid hydrogel formulations comprising of AMP loaded nanoparticles into hydrogel system have been explored to mitigate the limitations of NPs-based AMP delivery. Antimicrobial peptide loaded nanoparticles can be embedded within the hydrogel network using various methods, such as physical entrapment, covalent bonding, or electrostatic interaction. The choice of method depends on the type of nanoparticles and the hydrogel matrix used. The incorporation of antimicrobial peptide loaded nanoparticles into hydrogels can enhance their antimicrobial activity, overcome resistance, increase their stability, and provide sustained release of the peptides, which can reduce the frequency of administration and improve patient compliance.

Li *et al.*, developed an injectable *in situ* gel-forming system composed of human antimicrobial peptides 57 (AP-57) loaded nanoparticles and thermosensitive hydrogel to explore the potential application of antimicrobial peptides in cutaneous wound healing.^260^ The thermosensitive hydrogel encapsulated with nanoparticles was fabricated from biodegradable poly (l-lactic acid)-Pluronic L35-poly (l-lactic acid) (PLLA-L35-PLLA). In this study, hybrid hydrogel-based delivery system exhibiting high antioxidant and low toxicity demonstrated release of AP-57 for extended period in *in vitro* studies. Furthermore, the *in vivo* studies using AP-57-nanoparticles–hydrogel system indicated significant wound healing with wound closure of nearly 96.78 ± 3.12% along with enhanced granulation tissue formation, angiogenesis, and collagen deposition using full-thickness dermal defect model. In another study, hydrogel/liposome system for the photothermal triggered release of AMPs was developed to effectively treat Gram-negative *Pseudonomas aeruginosa* and Gram-positive *Staphylococcus aureus* infections.^165^ Herein, a poly (ethylene glycol) (PEG) hydrogel co-loaded with liposomes and gold nanorods (AuNRs) and AMP, IRIKIRIK-CONH2 (IK8) was employed for infection control. The photothermal triggered release of IK8 with laser irradiation at 2.1 W cm^−2^ for a period of 10 minutes caused heating to 55 °C indicating enhanced antimicrobial activity. The triggered release of IK8 at this laser intensity resulted in ∼6- and ∼7-log reductions in the number of *Pseudomonas aeruginosa* and *Staphylococcus aureus*, respectively. In addition, increased laser intensity 2.4 W cm^−2^, heating to 60 °C reported additional 5- and 2-log reductions in viable *P. aeruginosa* and *S. aureus* numbers, respectively. In a recent study, Zn^2+^ cross-linked alginate hydrogel incorporating RL-QN15 peptide loaded silica nanoparticles for chronic skin wound treatment.^166^ The slow and sustained release of loaded RL-QN15 ensured broad-spectrum antimicrobial activity in *in vitro* studies. Further analysis demonstrated improved healing of both methicillin-resistant *Staphylococcus aureus* biofilm-infected chronic wounds and full-thickness skin wounds in mice confirmed through accelerated re-epithelialization, granulation tissue formation, angiogenesis, and reduced inflammation. Recently, hybrid hydrogel systems-based wound dressings with feasibility of real-time diagnosis of infected wounds have been developed.^261,262^ Ran *et al.*, developed injectable theranostic hydrogels encapsulated with antimicrobial peptide ε-polylysine (ePL) and tetrakis(4-carboxyphenyl) porphyrin (TCPP)-loaded polydopamine (PDA) nanoparticles for real-time diagnosis of infected wounds, on-demand removal of bacterial debris, and imaging-guided antibacterial photodynamic therapy (PDT).^262^ The acid-triggered release TCPP restored the fluorescence emissions and provided real-time imaging of infected wounds under 410 nm illumination. Furthermore, illumination at 660 nm and 808 nm resulted in precise bacterial capture and bacterial debris removal, respectively. With the regular dressings changes accelerated wound healing was observed as revealed through enhanced collagen deposition, angiogenesis and regulation of inflammation and oxidative stress. Furthermore, a theranostic pH responsive hydrogels based on light-triggered gelation was developed for long term visual imaging of infected wounds and on-demand photodynamic therapy of wound infections without using any antibiotics.^261^ The bacterial infection-activated colour changes of hydrogels with pH change and illumination provided real time monitoring of infected wounds and wound healing process. The advantage of this hybrid hydrogel system includes strong bactericidal effect through photodynamically produced reactive oxidative species and no requirement of any additional photo initiators. Despite of its promising results, hydrogels encapsulating antimicrobial peptide loaded nanoparticles need optimization of utilized materials properties, including their mechanical strength, stability, and biocompatibility, to ensure their safety and efficacy for different applications. In addition, further studies are needed to investigate the potential of AMP to overcome of resistance in various bacterial strains in clinical studies.^259^ Overall, hydrogels incorporating antimicrobial peptide loaded nanoparticles are a promising approach that can potentially address the challenges associated with bacterial infections, MDR and improve patient outcomes.

## 5. Challenges in the clinical translation of nanoparticle-incorporated hydrogels

The emerging experimental research on nanoparticles-incorporated hydrogels (discussed in the above section) for antimicrobial effect demonstrates the potential of these systems in the delivery of antimicrobial agents, improving antimicrobial activity, and overcoming antibiotic resistance *in vitro* and *in vivo* (animal studies). In addition to the promising results of the experimental studies on the nanoparticle–hydrogel systems, the commercial availability of nanoparticle-containing antimicrobial dermal patches for wound healing are encouraging examples for the clinical translation of the antimicrobial nanoparticle–hydrogel systems.^263^ However, despite significant progress and promising results at the laboratory scale the clinical translation of these nanoparticle–hydrogel systems for delivery of the antimicrobial agents is a challenging task. Currently, no antimicrobial nanoparticle–hydrogel system is commercially available and only some such systems have entered clinical trials (mainly for local applications) such as silver nanoparticles containing xanthan gum hydrogel (NCT01442103), Silver nanoparticle gels for pain associated with a postoperative root canal (NCT03692286), Gold–silver-Cu_2_O nano gel with photothermal therapy (PTT) form antibiotic-resistant eye keratitis (NCT05268718), and PLGA nanoparticles coated with chitosan encapsulating ciprofloxacin incorporated into temperature-sensitive pluronic hydrogel treatment of endodontic infections (NCT05442736) (https://clinicaltrials.gov).^1,264,265^

The challenges faced in the clinical translation of the nanoparticle–hydrogel systems are related to both hydrogels and nanoparticles (independently and combined).^139^ The main challenges in clinical translation include the *in vivo* safety/toxicity, and clearance of the individual materials (both hydrogel and nanomaterial) especially after internal application.^264^ Although hydrogels are generally considered biodegradable and biocompatible but they can induce immune responses in the body due to prolonged exposure due to delayed clearance.^139^ Additionally, for injectable (*in situ*) nanoparticle-hydrogel gelation at the proper time is also critical to achieving optimal therapeutic effects at the desired area.^139^ These challenges related to hydrogels can be controlled by careful selection of the polymers and crosslinking techniques to control the gelation time and degradation rate of the hydrogels.^139^ On the other hand, the challenges faced in the clinical translation of the nanomaterials are more complicated due to their complex *in vivo* behaviours *i.e.* stability issues (aggregation) due to interaction with different proteins in biological systems, safety/toxicity problems especially chronic toxicity due to accumulation of the non-degradable nanoparticles and loss of desired targeting due to interactions *in vivo*.^264^ Most of these challenges are related to the physiochemical character of nanoparticles (chemical design, size, shape, charge, concentration, *etc.*) that should be fine-tuned to overcome these challenges.^263,264,266^ In addition, when the hydrogels and nanoparticles are combined as nanoparticle–hydrogel systems their safety evaluation and clinical translation becomes even more complex.^264^ To overcome these challenges of translation of these systems to clinics, the researchers should investigate in-depth the *in vivo* interaction of the system, their toxicity (acute and chronic), and the ultimate fate inside the body using a variety of advanced techniques.

## 6. Conclusion and future prospects

Nanoparticles incorporated hydrogels with the potential to enhance either mechanical stability or biological performance have gained significant attention in recent years, especially for the delivery of antimicrobial agents. The future prospects of these hydrogels are immense and promising, due to their unique properties such as high water content, biocompatibility, biodegradability, and drug release. One of the most significant advantages of using nanoparticles-incorporated hydrogels for drug delivery is the ability to control the release of the drug. The rate of drug release can be programmed depending on the desired therapeutic effect. This makes them an ideal platform for sustained delivery especially for chronic and long-term treatments. Apart from controlled drug release, nanoparticles incorporated in hydrogels also offer improved bioavailability, reduced side effects, and increased stability of the drug compounds and nanoparticles. They can also be engineered to target specific cells or organs, which further enhances therapeutic efficacy and reduces the likelihood of drug (antibiotic) resistance. In addition, nanoparticle-incorporated hydrogels are extremely versatile and can be tailored to meet specific drug delivery requirements such as stimuli-responsiveness. They can be easily modified to accommodate drug compounds of different sizes, charges, and solubilities. This makes them an ideal platform for delivering a wide range of antimicrobial agents, including antibiotics, antifungal agents, and antiviral drugs.

While significant progress has been made in recent years towards developing and optimizing nanoparticle-incorporated hydrogel materials for antimicrobial effect, there still remain a few challenges to improve their clinical applicability.^8^ The challenges lie in the comprehensive characterization of nanoparticles, hydrogel biomaterials, and implants at the level of *in vitro*, *in vivo*, and long-term clinical characterization to ensure safety and effectiveness. Some of the challenges of nanoparticle-hydrogel systems are:

Synthesis and fabrication: the methods for incorporating nanoparticles into hydrogels vary depending on the type, size, shape, and surface properties of the nanoparticles and the nature of the hydrogel network.^14^ The synthesis and fabrication techniques should ensure uniform distribution of nanoparticles within the hydrogel matrix, avoid aggregation or degradation of nanoparticles, and preserve the functionality and stability of both components.

Characterization: the characterization of nanoparticle–hydrogel systems requires multiple techniques to evaluate their physicochemical, mechanical, biological, and pharmacological properties. The characterization methods should be able to measure the interactions between nanoparticles and hydrogels, the distribution and release of nanoparticles from hydrogels, and the effects of nanoparticles and hydrogels on the surrounding environment and biological systems.^25^

Safety and biocompatibility: the safety and biocompatibility of nanoparticle–hydrogel systems depend on the properties of both components and their interactions with each other and with biological systems. The potential risks of nanoparticle–hydrogel systems include accumulation, toxicity, immunogenicity, inflammation, infection, and biodegradation.^34^ The safety, biocompatibility, toxicity, and fate of nanoparticle–hydrogel systems should be evaluated *in vitro* and *in vivo* using appropriate models and endpoints. Another major challenge, especially for clinical applications, includes clearance of the structure after the complete release of encapsulated therapeutic agents and immunogenic (foreign-body responses), which potentially limit the performance and clinical reformation of nanoparticle–hydrogels systems by forming collagenous capsules with prolonged retention. To circumvent this limitation, various new and emerging materials designs with superior capability such as ultra-low-fouling zwitterionic hydrogels should be employed to prevent capsule formation after implantation.^267^ Another, important challenge includes improving the ease of clinical utility of nanoparticle–hydrogels formulations. With continuous development in materials engineering and nanotechnology, nanoparticle–hydrogel system-based approaches are expected to generate new designs of biomaterials for the facilitation of therapeutic agents' delivery.

Overall, the future prospects of nanoparticle-incorporated hydrogels in the delivery of antimicrobial agents are promising. Their versatility, controlled release, and targeted drug delivery capabilities make them a valuable platform for developing novel drug delivery systems. As research in this field continues to advance, we can expect to see further improvements in the design and application of nanoparticle-incorporated hydrogels for drug delivery.

## Conflicts of interest

“The authors declare no conflicts of interest.”

## Supplementary Material
